# Specification and epigenomic resetting of the pig germline exhibit conservation with the human lineage

**DOI:** 10.1016/j.celrep.2021.108735

**Published:** 2021-02-09

**Authors:** Qifan Zhu, Fei Sang, Sarah Withey, Walfred Tang, Sabine Dietmann, Doris Klisch, Priscila Ramos-Ibeas, Haixin Zhang, Cristina E. Requena, Petra Hajkova, Matt Loose, M. Azim Surani, Ramiro Alberio

**Affiliations:** 1 School of Biosciences, University of Nottingham, Sutton Bonington Campus, Loughborough LE12 5RD, UK; 2 School of Life Sciences, University of Nottingham, Nottingham NG7 2RD, UK; 3 Wellcome Trust/Cancer Research UK Gurdon Institute, University of Cambridge, Tennis Court Road, Cambridge CB2 1QN, UK; 4 Department of Physiology, Development and Neuroscience, University of Cambridge, Downing Street, Cambridge CB2 3DY, UK; 5 MRC London Institute of Medical Sciences (LMS), London, UK; 6 Institute of Clinical Sciences (ICS), Faculty of Medicine, Imperial College London, London, UK; 7 Wellcome Trust Medical Research Council Stem Cell Institute, University of Cambridge, Tennis Court Road, Cambridge CB2 1QR, UK

**Keywords:** germ cells, DNA demethylation, pig, escapees, X-chromosome reactivation, single-cell RNA-seq, epigenetic resetting, transgenerational inheritance

## Abstract

Investigations of the human germline and programming are challenging because of limited access to embryonic material. However, the pig as a model may provide insights into transcriptional network and epigenetic reprogramming applicable to both species. Here we show that, during the pre- and early migratory stages, pig primordial germ cells (PGCs) initiate large-scale epigenomic reprogramming, including DNA demethylation involving TET-mediated hydroxylation and, potentially, base excision repair (BER). There is also macroH2A1 depletion and increased H3K27me3 as well as X chromosome reactivation (XCR) in females. Concomitantly, there is dampening of glycolytic metabolism genes and re-expression of some pluripotency genes like those in preimplantation embryos. We identified evolutionarily young transposable elements and gene coding regions resistant to DNA demethylation in acutely hypomethylated gonadal PGCs, with potential for transgenerational epigenetic inheritance. Detailed insights into the pig germline will likely contribute significantly to advances in human germline biology, including *in vitro* gametogenesis.

## Highlights

• Gene expression profiles of pig and human primordial germ cells are closely aligned
• Pre-migratory pig PGCs undergo DNA demethylation, XCR, and histone remodeling
• Identification of DNA demethylation-resistant loci in the pig germline

## Graphical Abstract

**Figure:**
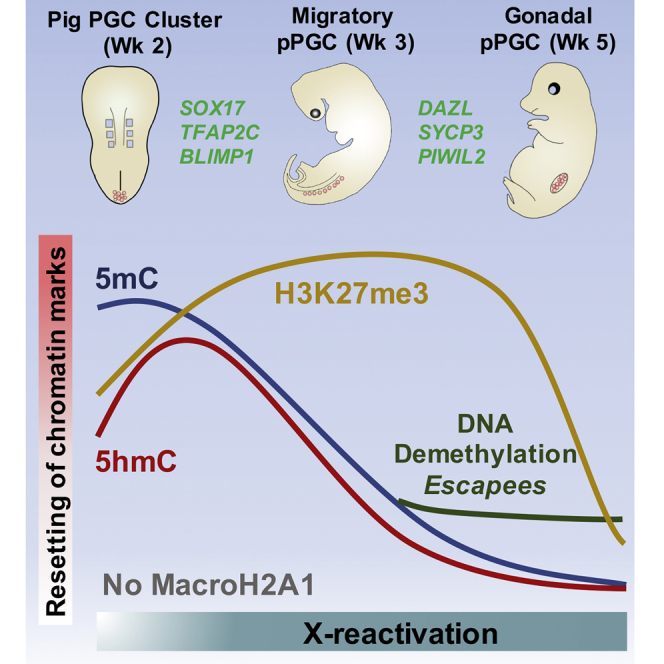

## Teaser

Zhu et al. show that pig primordial germ cells (PGCs) undergo DNA demethylation, histone remodeling, and X chromosome reactivation after specification. Pig PGCs retain few methylated loci after genome-wide demethylation, with potential for transgenerational inheritance. Species comparisons shows close similarities in transcriptional profiles of pig and human PGCs.

## Introduction

The germline transmits hereditary information, which ensures continuity of the species. Development of primordial germ cells (PGCs), the precursors of gametes, begins in peri-gastrulation embryos and is governed by a network of transcriptional regulators. Extensive epigenetic reprogramming follows, which includes erasure of imprints and, potentially, epimutations for restoration of totipotency (Hill et al., 2018; Kurimoto et al., 2008; Tang et al., 2016). Although the principles of mammalian germline development are emerging, so are some important differences and gaps in our knowledge (Kobayashi and Surani, 2018; Saitou and Miyauchi, 2016).

We have shown previously that the molecular program of pig PGCs (pPGCs) corresponds to what is known about human PGCs (hPGCs), indicating that studies in the pig may be informative for understanding the development of hPGCs (Kobayashi et al., 2017). A critical period of human germline development is between week 2 and week 4, when PGCs are specified and migrate toward the gonads (Leitch et al., 2013). However, human embryos are not accessible during these critical stages; consequently, we have little or no information about germline development during this period.

At the equivalent developmental period in pigs, pPGCs are specified between embryonic day 12 (E12)–E14, following sequential upregulation of SOX17 and BLIMP1 in response to BMP signaling (Kobayashi et al., 2017), as is the case during induction of hPGC-like cells (hPGCLCs) *in vitro* (Irie et al., 2015). pPGCs commence migration at ∼E15 through the hindgut until they reach the gonadal ridges by E22 and undergo extensive proliferation between E28–E42 (Hyldig et al., 2011a, 2011b).

Shortly after pPGC specification, pre-migratory pPGCs display initiation of epigenetic reprogramming, characterized by global reduction in DNA methylation and H3K9me2 (Hyldig et al., 2011a; Kobayashi et al., 2017; Petkov et al., 2009). Upon colonization of the gonads, pPGCs show asynchronous demethylation of imprinted genes and retrotransposons (Hyldig et al., 2011a, 2011b; Petkov et al., 2009). Accordingly, there is protracted epigenetic reprogramming in the pig germline over a period of several weeks.

Studies of early hPGCs have relied on pluripotent stem cell-based *in vitro* models, which showed that hPGCLCs originate from cells with a posterior primitive streak (PS)/incipient mesoderm-like identity following exposure to BMP, revealing SOX17 to be a critical determinant of the PGC fate (Irie et al., 2015; Kojima et al., 2017). Studies of *ex vivo* hPGCs showed that epigenetic reprogramming in the human germline is also protracted and asynchronous compared with mice (Gkountela et al., 2015; Guo et al., 2015; Tang et al., 2015), but there is limited scope for detailed investigations of *ex vivo* human embryos. We posit that investigations in the pig that develop as bilaminar discs, unlike egg cylinders of laboratory rodents, might provide insights into fundamental mechanisms of germline development that would apply widely to non-rodents, including the human germline.

Here, using single-cell transcriptome (single-cell RNA sequencing [scRNA-seq]) and whole-genome bisulfite sequencing (WGBS), we reveal the transcriptional program and epigenetic features of pPGCs during a critical interval of development that is largely inaccessible for humans. We observed a close transcriptional alignment between pPGCs and hPGCs. We also observed extensive epigenetic reprogramming characterized by DNA demethylation, X chromosome reactivation (XCR) and histone modifications in pre- and early migratory pPGCs. Metabolic dampening of glycolytic metabolism genes and the reactivation of some pluripotency-associated genes accompanied these events. We identified genomic loci escaping global DNA demethylation, with potential for transgenerational epigenetic inheritance.

## Results and discussion

### Single-cell profiling of pPGCs

**Figure 1:**
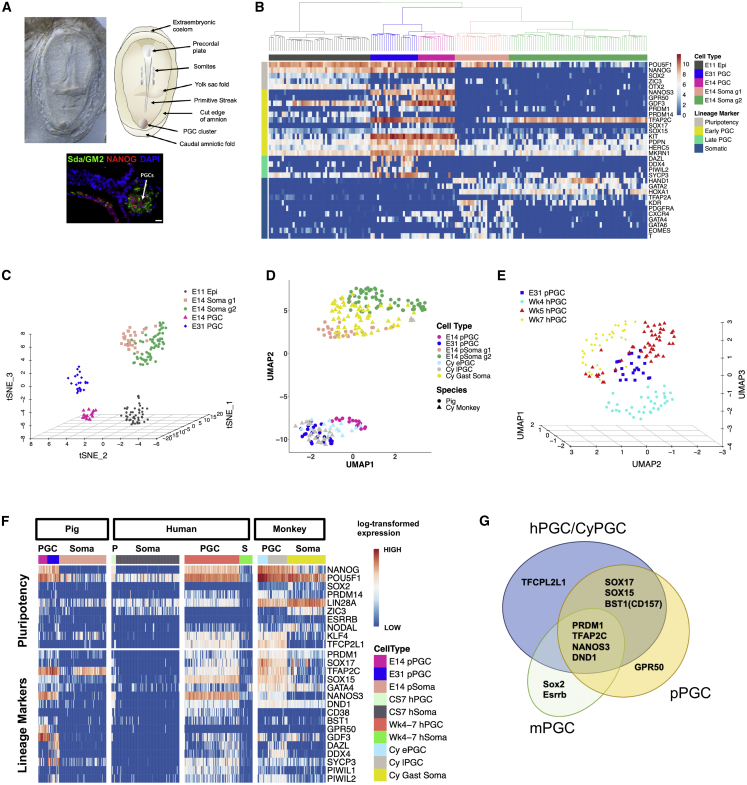
Transcriptional profile of pPGCs and comparison with hPGCs (A) Bright-field top view image of a pig embryo (left) and diagrammatic representation (right) showing key structures. Bottom image: IF staining of a midline sagittal section of an E14 embryo, showing a PGC cluster (white arrow) in the caudal end. Scale bar, 20 μm. (B) UHC clustering of all expressed genes. A subset of selected marker genes was used for the heatmap. Color scale unit, log-transformed transcripts per kilobase million (TPM). (C) t-SNE showing relationships between E11 Epi cells, E14 somatic cells, and E14 and E31 PGCs. (D) UMAP plot showing integration of cyPGCs (E13-55) and pPGCs (E14-31) and somatic cells. (E) UMAP plot showing integration of hPGCs (weeks 4–7) and pPGCs (week 5). (F) Expression profiles of pluripotency genes and lineage markers in pPGCs, hPGCs, cyPGCs, and somatic cells. Wk4-7: Weeks 4–7; cy ePGCs: early cyPGCs (E13–E20); Cy lPGCs: late cyPGCs (E36–E55); cy gast soma: cy gastrulating cells (E13–E20); CS7: Carnegie stage 7; S, soma; P, PGCs. (G) Schematic highlighting species differences in expression of key PGC genes. See also Figures S1 and S2 and Tables S1 and S4.

pPGCs first emerge in E12 embryos, forming a cluster of ∼60 cells that expands to ∼150–200 by E14 (Kobayashi et al., 2017). To investigate the transcriptome of pre-migratory pPGCs, we dissected the posterior region of E14 embryos. We also isolated germ cells from E31 gonads (Table S1). After dissociation of the tissues into single cells and fluorescence-activated cell sorting (FACS) using an anti-Sda/GM2 antibody (Klisch et al., 2011), we manually picked individual cells for analysis (Figure 1A; Figure S1A). We obtained scRNA-seq data of 17 Sda/GM2^+^ cells (pre-migratory pPGCs) and 89 Sda/GM2^−^ (surrounding cells) from E14 embryos. We similarly analyzed 22 Sda/GM2^+^ early (E31) gonadal PGCs using the Smart-Seq2 protocol (Picelli et al., 2014). After sequencing, we identified closely related cells using unsupervised hierarchical clustering (UHC) and t-stochastic neighbor embedding (t-SNE) analysis, including a dataset of pig E11 epiblasts (Ramos-Ibeas et al., 2019; Figures 1B and 1C). Epiblast (Epi) cells and E14 surrounding somatic cells clustered separate from pPGCs (Figures 1B and 1C). In E14 and E31 pPGCs, we detected *PRDM1* (*BLIMP1*), *TFAP2C*, *NANOS3*, and *KIT* and high expression of the pluripotency genes *NANOG* and *POU5F1*. The late PGC markers *DAZL*, *DDX4*, and *PIWIL2* were only detected in E31 gonadal PGCs. We did not detect *SOX2* in most (33 of 39) pPGCs. Of the six *SOX2*-positive cells, four did not express *SOX17*, suggesting a mutually exclusive expression profile between *SOX2* and *SOX17* during pPGC specification. We found expression of *PDPN*, *HERC5*, and *MKRN1* (Figure 1B), which has been reported recently in early hPGCs from a rare gastrulating Carnegie stage 7 (CS7) human embryo (Tyser et al., 2020). SOX17 protein was present in pre-migratory and gonadal pPGCs, as observed by immunofluorescence (IF) (Figure 1A; Figure S1A), although the *SOX17* transcript was found in a subset of pPGCs (6 of 17 in E14 and 12 of 22 in E31 pPGCs) (Figure 1B). Interestingly, low and fluctuating *SOX17* expression is also observed in early hPGCs in CS7 human embryos, whereas the endoderm lineage shows consistent and high SOX17. Low and fluctuating SOX17 expression in early pPGCs and hPGCs might reflect a conserved mechanism to regulate gene dosage to prevent expression of endoderm genes in hPGCs and pPGCs (Irie et al., 2015; Kobayashi et al., 2017; Tyser et al., 2020).

The posterior somatic cells in E14 embryos, which are likely neighbors of pPGCs, segregated into two clusters: E14 soma g1 and E14 soma g2 (Figure 1B; Figure S1B). In E14 soma g1 cells, we observed high expression of the PS and embryonic mesoderm genes *T*, *EOMES*, and *MESP1*; the cell surface markers *KDR*, *PDGFRA*, *CXCR4*, and CD13 (*ANPEP*) (Kopper and Benvenisty, 2012); and the signaling components *WNT5A*, *WNT8A*, and *LEF1*. These cells also showed high levels of *SNAI1*, *ZEB2*, and *CDH2* (N-Cadherin) and low expression of *CDH1* (E-cadherin). The gene expression profile in soma g1 cells suggests that these cells may be undergoing epithelial-mesenchymal transition (Pan et al., 2016: Stemmler et al., 2019). In contrast, soma g2 cells in E14 embryos exhibit epithelial features with hallmark expression of the amnion-specific genes *GATA3*, *GATA2*, *TFAP2A*, *TFAP2C*, *OVOL1*, and *KRT7/8/1*8 (Gomes Fernandes et al., 2018; Xiang et al., 2020) as well as the cell adhesion-related genes *ITGA3*, *PKP2*, *PODXL*, and *AHNAK* (Saykali et al., 2019). Trajectory analysis confirmed the pseudo-temporal relationship among these cells, with soma g1 nascent mesoderm being closer to Epi cells, whereas soma g2 diverge from g1 and PGCs (Figure S1C). There is evidently a close spatial relationship between pre-migratory pPGCs, mesoderm, and amnion precursors (see below). Previous studies have shown that, after their induction in posterior early-PS Epis, the PGC cluster localizes at the embryonic and extraembryonic border in pig pre-somatic-stage embryos (Kobayashi et al., 2017; Wolf et al., 2011). Similarly, in a gastrulating CS7 human embryos, hPGCs have been suggested to emerge from the PS and are set apart from nascent mesoderm and other lineages (Tyser et al., 2020). Importantly, these cell types are induced by BMP signaling, which is detected in the posterior end of the pig embryo from E12 onward (Valdez Magaña et al., 2014; Yoshida et al., 2016).

Next, we identified differentially expressed genes (DEGs) between E14 PGC and E14 somata (g1 and g2 combined) and found enrichment in PGCs for “germ cell development” and “positive regulation of double-strand break repair” by Gene Ontology (GO) analysis (Figure S1D; Table S2), indicating the importance of DNA repair during pre-meiotic PGC development (Guo et al., 2015; Hajkova et al., 2010; Hill and Crossan, 2019). Furthermore, GO analysis showed reduced expression of glycolysis-associated genes in PGCs between E14 and E31 and upregulation of genes controlling mitochondrial activity and oxidative phosphorylation (Figures S1E and S2A; Table S2). An increase in mitochondrial activity in E31 gonadal PGCs is also suggested by higher expression of mtDNA-encoded genes compared with E14 (Figure S1E). Thus, these results are consistent with a metabolic shift in pPGCs during their migration and epigenetic resetting, as reported previously for gonadal mouse PGCs (Hayashi et al., 2017) and hPGCs (Floros et al., 2018). Notably, the expression changes of metabolic genes start in pre-migratory pPGCs, supporting previous observations in hPGCLCs (Tischler et al., 2019).

To gain insight into the signaling microenvironment of the posterior end of E14 pig embryos, we analyzed the expression profile of genes involved in different signaling pathways. GO terms and KEGG pathway analysis of E14 somatic compartment showed enrichment for WNT, BMP, transforming growth factor β (TGF-β), and phosphatidylinositol 3-kinase (PI3K)-akt signaling (Figures S1D and S2B), similar to findings from pre-streak and early-PS pig embryos (E10.5–E12.5) (Valdez Magaña et al., 2014; Yoshida et al., 2016). Previous work showed that WNT signaling confers to pig germ cell precursors the competence to respond to BMP and triggers the germ cell program at around E12 (Kobayashi et al., 2017; Kojima et al., 2017). We show that, after onset of pPGC specification, these key signaling molecules are still expressed in this area of the extraembryonic mesoderm, which gives rise to amnion (Perry, 1981).

In contrast to the soma, from the earliest developmental stage (E14), pPGCs showed upregulation of Jak/STAT-insulin pathways genes (Figure S2B), which is consistent with the described function of LIF as a survival factor in PGCs (Hayashi et al., 2011; Ohinata et al., 2009).

We next examined the cell cycle stage of pre-migratory pPGCs and determined that more than 85% of cells were in the G1 or G2 cell cycle stage, in contrast to their early gonadal counterparts, which were mostly in S phase (46%) (Figure S2C). These findings are in line with previous observations showing no EdU incorporation in E14 pPGCs and a high proportion of E17 pPGCs arrested in G2, suggesting that pre-gonadal PGCs do not proliferate rapidly (Hyldig et al., 2011a; Kobayashi et al., 2017). These kinetics are also consistent with limited proliferation of hPGCLCs during the first days (day 4) of development, which then resumes during extended culture (Gell et al., 2020).

### Surface markers in pPGCs

Membrane proteins participate in numerous cellular processes, such as cell signaling, transport, and migration. Therefore, we sought to identify pPGC-specific membrane proteins by selecting pPGC-specific genes with relevant GO terms and/or those that are curated in the Cell Surface Protein Atlas (Bausch-Fluck et al., 2015). As reported before for hPGCs and early cynomolgus monkey PGCs (cyPGCs) (Gomes Fernandes et al., 2018; Sasaki et al., 2016; Tang et al., 2015), *KIT* and *PDPN* were upregulated in pre-migratory pPGCs (Figure S2D). We also determined expression of the orphan receptor *GPR50*, which is specific for early but not gonadal pPGC (Figure S2E). GPR50, known to heterodimerize with surface receptors of the TGF-β family, was detected on the cell membrane of early migratory pPGCs and in the nucleus of gonadal PGCs (Figure S2F). The nuclear localization is indicative of cleavage of the C terminus following heterodimerization. GPR50 has been shown to promote cell migration and to decrease TGF-β-driven cell proliferation (Wojciech et al., 2018). Expression of GPR50 in E17 pPGCs coincides with their migration to the gonads and reduced cell cycle progression (Figure S2C). We also detected high levels of *CXCR4*, needed for PGC migration in mice (Molyneaux et al., 2003), in E14 pPGCs suggesting onset of migration (Figure 1B). GDF3, a mammal-specific TGF-β ligand expressed in cyPGCs (Sasaki et al., 2016) and gonadal hPGCs (Li et al., 2017), is also enriched in early pPGCs (Figure S2D). The CD markers *CD126* (*IL-6R*) and *CD157* (*BST1*), closely related to the hPGC marker *CD38*, and the orphan receptor *GPR133* (*ADGRD1)*, which is also expressed in hPGCs, are upregulated in pPGC (Figures S2D and S2E). Additionally, upregulation of *SLC23A2* in pre-migratory PGCs may contribute to cellular uptake of vitamin C and promote TET1 activity in PGCs (DiTroia et al., 2019). The surface molecules identified depict a profile of cells preparing to embark on their migration toward the gonad and onset of epigenetic resetting.

### A conserved transcriptional program between pPGCs, hPGCs, and cyPGCs

To investigate the conservation of germline development in detail, we compared the expression profiles of pPGCs, hPGCs, and cyPGCs by integrating scRNA-seq datasets (Li et al., 2017; Sasaki et al., 2016; Tyser et al., 2020). Pre migratory (E14) pPGCs cluster with E13–E20 cyPGCs (ePGCs), whereas E31 pPGCs clustered with E36–E55 cyPGCs (lPGCs) (Figure 1D). Similarly, tight clustering was determined between E14 pPGCs and CS7 hPGCs in ∼E19 human embryos (Figure S1F). Gonadal E31 (week 5) pPGCs clustered with week 5 hPGCs (Figure 1E). hPGCs, pPGCs, and cyPGCs show similar expression profiles of key germline genes (*SOX17*, *PRDM1* [*BLIMP1*], *TFAP2C*, *NANOS3*, and *DND1*) and pluripotency genes (*NANOG*, *POU5F1*, and *LIN28A*) (Figure 1F; Table S3). As in human and cyPGCs, the endoderm marker *GATA4* is also widely expressed in pPGCs, the mesoderm marker *T* (*BRACHYURY*) is expressed in early pPGCs and maintained in some gonadal pPGCs, and *EOMES* is absent from pre-migratory cells (Figure 1B). Conversely, the naive pluripotency gene *TFCP2L1* is not detectable in pPGCs, in contrast to human and cyPGCs (Figure 1F). *KLF4*, which is not detected in early hPGCs and is found at variable levels in cyPGCs, is expressed in few pPGCs. A recent study shows that both genes may be dispensable for hPGCLC specification (Hancock et al., 2020); however, further work is needed to establish the role of these naive pluripotency genes in germline development. Similarly, *PRDM14*, which is not detected in CS7 hPGCs (Tyser et al., 2020), is only detectable in some gonadal pPGCs, suggesting that it may not have an essential role during pPGC specification (Figures 1B and 1F; Kobayashi et al., 2017). Recent evidence shows that *PRDM14* may have a role in maintenance of hPGCs after specification (Sybirna et al., 2020).

This analysis shows that expression of critical transcription factors involved in pPGC specification are largely equivalent to that of hPGCs and cyPGCs but differs from that of mice (Figure 1G; Figure S1G; Guo et al., 2015; Irie et al., 2015; Kojima et al., 2017; Sasaki et al., 2016). Although the basis of the transcriptional divergence is not fully understood, it is noteworthy that pigs and humans (and most other mammals) develop a bilaminar disc prior to onset of gastrulation, whereas some rodents, like mice and rats, have evolved an egg cylinder. The divergence in development and molecular aspects, such as the pluripotency network, which may facilitate evolution of embryological innovations, merits further consideration (Johnson and Alberio, 2015).

The reduced expression (*KLF4* and *PRDM14*) or lack of expression (*SOX2* and *TFCP2L1*) of some of these genes in the pig germline prompted us to investigate the underlying pluripotency features of pPGCs in more detail. We created signature gene sets from E6 ICM as well as E8 and E11 Epis (Ramos-Ibeas et al., 2019) and examined their expression in pre-migratory (E14) and gonadal pPGCs (E31). A strong pig E8 Epi signature score was determined for both (E14 and E31) pPGC stages, with gonadal pPGCs showing a stronger ICM signature score compared with E14 pPGCs (Figures S1G and S1H; Table S4). The signature genes contributing to these scores include elevated expression of well-known transcription factors (*POU5F1*, *NR5A2*, and *SOX15*) but also of chromatin-related genes (*HELLS*, *BRDT*, and *ZAR1*) and regulators of transposable element activity (*MOV10*, *ASZ1*, *PLD6*, *HENMT*, *TDRKH*, and *SAMHD1*), indicating that restoration of a gene signature common with ICM/E8 Epi in early PGCs is linked to epigenetic resetting of the germline, which does not occur in the neighboring somatic lineages.

### Onset of DNA demethylation in pre-migratory pPGCs

**Figure 2:**
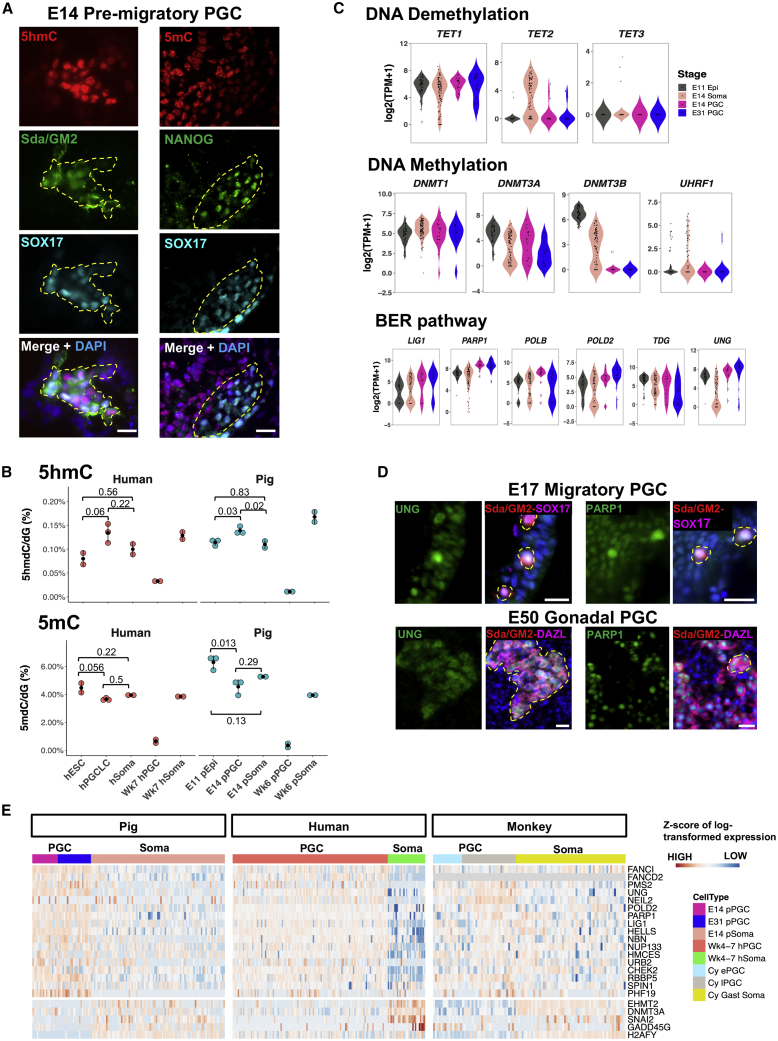
Active DNA demethylation in pre-migratory pPGC. (A) IF staining for 5hmC and 5mC in a E14 PGC cluster (yellow dashed lines). PGCs are marked by SOX17, Sda/GM2, and Nanog. Scale bar, 20 μm (B) 5hmC and 5mC levels determined by LC-MS/MS. Methylation levels are indicated relative to total levels of deoxyguanine (dG). The p values are based on combined ANOVA and Holm’s post hoc test. Data points indicate biological replicates. (C) Expression of epigenetic modifiers for DNA methylation/demethylation and BER pathway components in E11 Epis, E14 somata, and E14 and E31 PGCs. (D) IF staining for UNG and PARP. The yellow circle marks PGCs. Scale bar, 20 μm. (E) Expression heatmap of epigenetic modifiers differentially expressed in pPGCs, hPGCs, and cyPGCs compared with somata. Cy ePGC, early cyPGC (E13–E20); Cy lPGC, late cyPGC (E36–E55); Cy Gast soma, cynomolgus monkey gastrulating cells (E13–E20). Gray color in the heatmap indicates not available. *Z* scores of log-transformed matrices were used. Because different expression units are used for each species, values in the color scale are replaced by HIGH and LOW. See also Figure S3.

Next, we investigated the onset of epigenetic reprogramming in pPGC using a combination of approaches. Analysis by IF showed 5-hydroxymethylcytosine (5hmC) staining in E14 pPGCs concomitant with reduced 5-methylcytosine (5mC) (Kobayashi et al., 2017), suggesting onset of DNA demethylation (Figure 2A). Quantification of 5mC and 5hmC using liquid chromatography-tandem mass spectrometry (LC-MS/MS) (Hill et al., 2018) was consistent with the IF data, demonstrating that 5hmC levels are higher in pre-migratory (E14) pPGC compared with the surrounding cells and Epis. Conversely 5mC levels were lower in pre-migratory pPGC compared with Epis (Figure 2B). DNA methylation reaches the lowest levels in gonadal pPGCs (Figure 2B; Figure S3B). Importantly, we also determined similar kinetics of 5mC and 5hmC in D4 hPGCLCs and equivalent human gonadal samples (Figure 2B), in accordance with previous reports of early gonadal hPGCs (Guo et al., 2015; Tang et al., 2015). Coupled with the high levels of 5hmC, we detected a sharp decline in *DNMT3B* and *UHRF1*, indicating that the methylation machinery is downregulated from the pre-migratory stage and persists until gonadal stages (Figure 2C; Figure S3A).

We also found that multiple base excision repair (BER) pathway genes (*LIG1*, *POLD2*, *POLB*, *PARP1*, and *UNG)* were upregulated in E14 and E31 pPGCs (Figures 2C and 2D), supporting the suggestion that active removal of TET-oxidized products in PGCs may be mediated by the BER pathway (Hackett et al., 2013; Hajkova et al., 2010; Hill et al., 2018). Furthermore, upregulation of “readers” for TET-oxidized products (*HELLS*, *HMCES*, *NUP133*, and *URB2*) (Spruijt et al., 2013) was observed in pPGCs, cyPGCs, and hPGCs, suggesting that 5hmC may be a dynamic and functional marker in early PGCs (Figure 2E). In addition to the BER pathway, we detected upregulation of components of Fanconi anemia (FA) (*FANCI* and *FANCD2*), mismatch repair (*PMS2*), and double-strand break repair (*NBN*) pathways in pPGCs, indicating that multiple DNA repair mechanisms may be activated during epigenetic reprogramming of pre-migratory PGCs (Figure 2C; Figure S2E). Our data from IF, LC-MS/MS, and scRNA-seq show that non-replicative pre-migratory pPGCs initiate TET activities and activate BER pathway components potentially mediating active DNA demethylation, followed by passive demethylation in migratory and gonadal PGCs, as shown by the reduction in DNMT3A/B and UHRF1. These observations suggest that DNA demethylation is mediated by active and passive mechanisms that start in early PGCs (E14), which reach the lowest levels in gonadal stages. These mechanisms cannot be studied in human nascent PGCs, but our findings in the pig concur with those reported previously showing limited DNA replication (Gell et al., 2020) and high levels of 5hmC in D4 hPGCLCs cells (Tang et al., 2015) and increased expression of BER pathway genes in week 4 hPGCs (Guo et al., 2015).

The extended DNA demethylation kinetics in the pig (∼21 days) contrasts with the rapid demethylation in mouse PGCs (∼5 days), where it is primarily mediated by passive demethylation during early migration, followed by active and passive demethylation in the gonads (Hackett et al., 2013; Hill et al., 2018; Kagiwada et al., 2013). The protracted process in the pig germline reflects the longer period of development of pPGCs and hPGCs, which are specified around week 2 and reach the gonadal ridges at weeks 4 and 5, respectively (Takagi et al., 1997; Witchi, 1948); in the mouse, this process takes ∼4 days (from E6.25–E10.5). However, the number of PGCs in the early gonad is similar between species: ∼2,600 in mouse E11.5 (Kagiwada et al., 2013), ∼3,000 in week 5 human male fetal gonads (Bendsen et al., 2003), and ∼3,000–5,000 in pig week 4 gonads (Black and Erickson, 1968; unpublished data). To reach the same number of gonadal germ cells, mouse PGCs proliferate faster and divide approximately every 12 h, whereas hPGCs divide every 6 days (Bendsen et al., 2006; Kagiwada et al., 2013). Thus, in the context of prolonged doubling times in hPGCs and pPGCs, complementary DNA demethylation mechanisms (active and passive) apparently ensure efficient initiation of DNA methylation reprogramming.

### Dynamic chromatin changes in pPGCs

**Figure 3:**
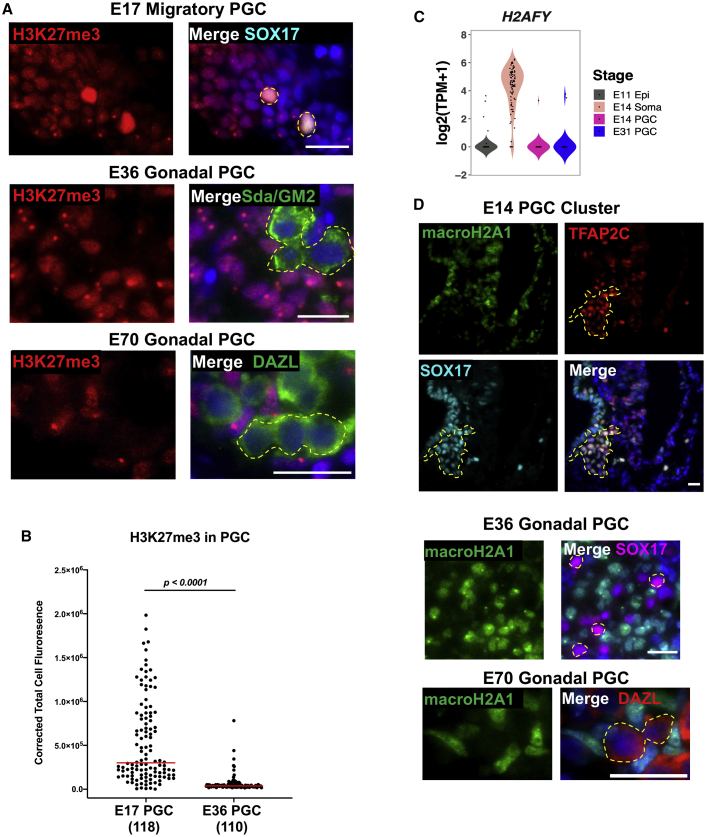
Histone remodeling in pre-migratory, early migratory, and gonadal pPGCs (A) H3K27me3 IF in pPGCs. Yellow dashed lines mark PGCs. Scale bar, 20 μm. (B) Quantification of H3K27me3 in PGCs. The red line indicates the median value. Significance was determined by Mann-Whitney *U* test. (C) Violin plot showing expression of *H2AFY*. (D) IF of macroH2A1. Scale bar, 20 μm. PGCs are shown by yellow dashed lines. See also Figure S3.

**Figure 4:**
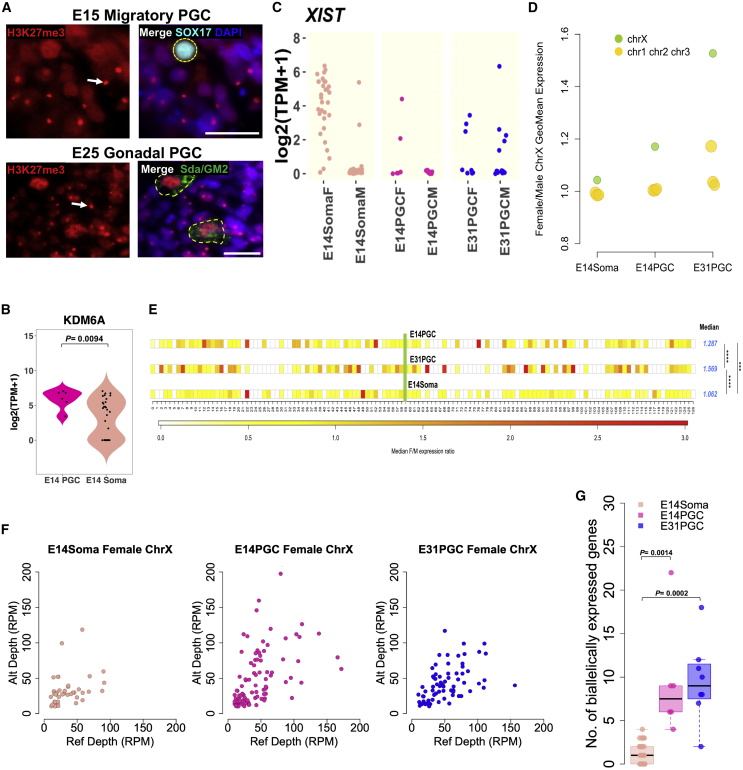
XC reactivation in pre-migratory pPGCs (A) IF staining for H3K27me3. Xi-associated H3K27me3 is detected in somatic cells (arrows). The yellow dashed circle marks PGCs. Scale bar, 20 μm. (B) Expression of *KDM6A* in E14 cells. The p value was determined by Mann-Whitney *U* test. (C) Expression of *XIST* in E14 somatic cells and E14 and E31 PGCs. F, female; M, male. (D) Female-to-male expression ratio of XC genes versus autosomes (chr1, chr2, and chr3) in E14 somatic cells, E14 PGCs, and E31 PGCs. (E) Median female-to male-expression ratio across XC. The p values (^∗∗∗^p < 0.001, ^∗∗∗∗^p < 0.0001) were determined by pairwise Wilcoxon test. Presumed XCI is indicated in green. (F) Biallelically detected SNPs on XC genes. Each dot represents one biallelically detected SNP. x axis, sum of reads (RPM) that are mapped to the reference alleles; y axis, sum of reads (reads per kilobase million [RPM]) that are mapped to the alternative alleles. (G) Number of biallelically expressed genes. The p value was determined by Kruskal-Wallis test followed by Dunn’s test. See also Figure S4.

We next examined chromatin features of pPGCs as part of epigenetic resetting and DNA demethylation in pPGCs. Although, overall, H3K27me3 was elevated in migratory (E17) and early gonadal (E25) PGCs, it decreased sharply in mid- and late gonadal PGCs (Figures 3A, 3B, and 4A), consistent with high expression of the *Polycomb-related complex 2* (PRC2) members *EZH2*, *SUZ12*, and *EED* in migratory and early gonadal pPGCs (Figure S3C). Furthermore, the PRC2-associated cofactor *PHF19*, required for PRC2 recruitment and activity (Ballaré et al., 2012), was enriched in early pPGCs (Figure S3C). Changes in other histone and chromatin remodelers were also detected, such as upregulation of components of the MII complex (*DPY30* and *RBBP5*) and the SWI/SNF proteins *SMARCA5* and *HLTF* (Figure S3C). Similar observations have been reported in week 4 hPGCs and D4 hPGCLCs (Gell et al., 2020; Gkountela et al., 2013; Gomes Fernandes et al., 2018; Tang et al., 2015). In contrast, mouse PGCs show persistent H3K27me3 in gonadal PGCs (Chuva de Sousa Lopes et al., 2008; Seki et al., 2005), which might have a role in maintaining genomic integrity during the period of active DNA demethylation (Liu et al., 2014). The decrease in H3K27me3 in gonadal pPGCs and hPGCs during extensive DNA demethylation suggests the possible existence of additional mechanisms that warrant future investigation.

MacroH2A1, the macro-histone variant encoded by *H2AFY* associated with H3K27me3 on developmental genes, was upregulated in somatic cells, but not in pPGCs, where it would act as a barrier to transcription factor-induced reprogramming (Gaspar-Maia et al., 2013; Figures 3C and 3D). MacroH2A1.1 modulates PARP1 activity and mediates the cellular DNA damage response (Posavec Marjanović et al., 2017). Interestingly, we found high PARP1 levels in pre-migratory and gonadal PGCs (Figures 2C–2E), suggesting that macroH2A depletion from early PGCs might contribute to maintenance of a chromatin configuration that facilitates the onset of epigenetic reprogramming. Consistent with the findings in pPGCs, *H2AFY* is downregulated in hPGCs and cyPGCs (Figure 2E). Furthermore, gonadal hPGCs have been shown to lack the closely related macroH2A2 (Tang et al., 2015).

### Extensive X chromosome reactivation in pre-migratory pPGCs

To gain further insights into reprogramming in pre-migratory pPGCs, we combined IF and transcriptomics analysis of XCR, which is characterized by loss of H3K27me3 enrichment on the inactive X chromosome (Xi) and bi-allelic expression of X-linked genes (Sugimoto and Abe, 2007). We found that, in pre/early migratory (E14–E17) and gonadal female pPGCs (E25), over 70% of cells showed faint or no H3K27me3 “spots” (Figure 4A; Figures S4C and S4D), suggesting XCR had already started in pre-migratory cells. Notably, the histone demethylase *KDM6A*, which is associated with loss of H3K27me3 in the inactive X chromosome (XC) (Borensztein et al., 2017; Mansour et al., 2012), was upregulated in E14 female PGCs (Figure 4B). To further analyze XCR at the transcriptional level, we measured *XIST* expression, which is critical for X inactivation (Jonkers et al., 2008). After determination of the sexual identity of E14 and E31 cells based on the cumulative levels of Y chromosome genes per cell (Figure S4A), we determined a reduction in *XIST* expression in the majority of E14 (4 of 6) and E31 (5 of 8) female pPGCs but not in female somatic cells (Figure 4C). *XIST* expression was also determined in some male gonadal PGCs, consistent with previous findings in hPGCs (Li et al., 2017; Vértesy et al., 2018). Furthermore, XC but not autosome expression in female E14 PGCs was significantly higher compared with male pPGCs, increasing further in E31 female PGCs (Figure 4D). In contrast, no gender differences were detected for XC or autosome expression in somatic cells (Figure 4D). At the single-cell level, the XC expression to total autosomal expression (X:allA) ratio was above 1 in all E31 female PGCs and most E14 female PGCs (Figure S4B), consistent with observations in female gonadal mouse PGCs (mPGCs) and hPGCs (Sangrithi et al., 2017). We also found no apparent relationship between X-linked gene reactivation and proximity to the XC inactivation (XCI) center (Figure 4E)

To rule out the possibility that the increased X:allA ratio and F:M ratio for XC were due to expression changes in one active XC instead of biallelic expression from both XCs, we analyzed gene expression at allelic resolution. E14 female somatic cells have a lower number of bi-allelic single-nucleotide polymorphisms (SNPs) (Figure 4F), which are likely to be genes that escape XCI in the pig. Studies show that 4%–8% and 15%–25% of X-linked genes in mice and humans, respectively, escape XCI to some degree (Carrel and Willard, 2005). These genes, which we called XC “escapers*”* to distinguish them from the DNA methylation escapees (see below), vary largely between tissues and species and have not been characterized in the pig. Therefore, we categorized X-linked genes containing biallelic SNPs in pig somatic cells as our XC escapers. Consistent with the increased X:A and F:M ratio, E14 and E31 female PGCs have a large number of non-escaper, biallelic SNPs, providing evidence of onset of XCR in pre-migratory PGCs (Figure 4F). We then identified biallelically expressed X-linked genes in female cells and found that all female pPGCs contain at least one biallelically expressed X-linked gene that is not found in somatic cells. In contrast to the sharp increase in biallelic gene expression, which is only detected in gonadal mPGCs (Sugimoto and Abe, 2007), pig pre-migratory and gonadal PGCs have higher numbers of biallelically expressed genes, suggesting that XCR is a cell-autonomous and asynchronous process taking place over a long period (Figure 4G).

Consistent with our findings in pPGCs, hallmarks of XCR have also been reported in hPGCs, showing loss of the H3K27me3 spot in week 4 (Tang et al., 2015) and biallelic expression of X-linked genes in week 7–8 PGCs; however, data from earlier stages are not available (Sangrithi et al., 2017; Vértesy et al., 2018). Even though it is not currently possible to conclude whether human XCR occurs as early as shown in pPGCs, our evidence of XCR in pre-migratory pPGCs contrasts with observations in mPGCs, where there is limited loss of H3K27me3 (<10%) and *Xist* (<15%) expression in pre-migratory PGCs; the increase in the X:A ratio is first detected in E11.5 PGCs (Chuva de Sousa Lopes et al., 2008; Sangrithi et al., 2017; Sugimoto and Abe, 2007). Our findings show that XCR begins in pre/early migratory pPGCs and continues in gonadal pPGCs.

### The DNA methylation level reaches the basal level in gonadal pPGC

**Figure 5:**
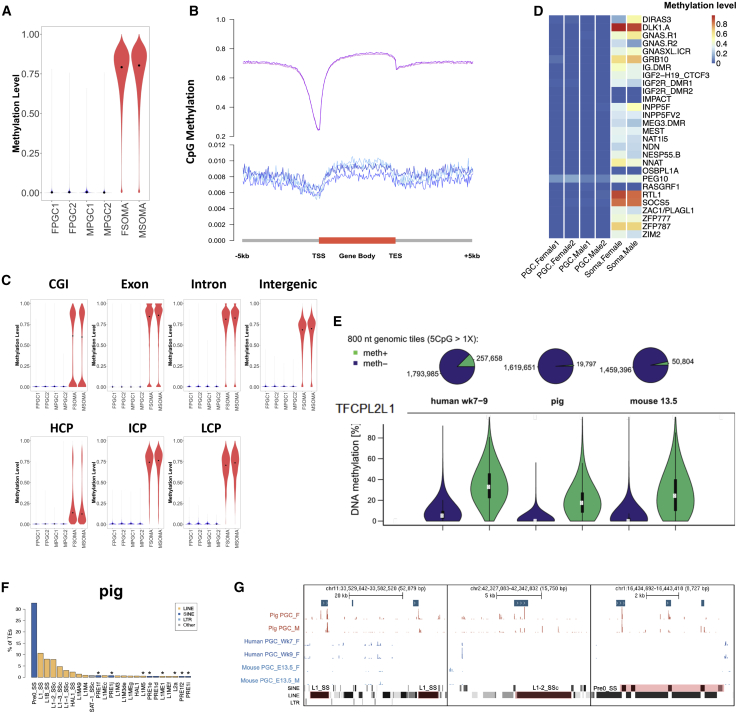
PBAT reveals the basal level of methylation in gonadal pPGCs (A) CpG methylation levels in 1-kb genomic tiles of week 5 (E35) female and male pPGCs and gonadal somatic cells. Black points indicate the median. (B) Averaged CpG methylation level profiles of all genes from 5 kb upstream (−) of transcription start sites (TSSs) through scaled gene bodies to 5 kb downstream (+) of transcription end sites (TESs). Different y axes are used for pPGCs and somatic cells because of the extremely low level of methylation in pPGCs. (C) Violin plots showing CpG methylation levels in different genomic features. (D) CpG methylation levels of imprinted regions in pPGCs and somata. (E) Top: proportion of demethylated loci (meth−) and demethylation-resistant loci (meth+) in week 5 pPGCs, week 7–9 hPGCs, and E13.5 mPGCs (the number of meth+ and meth− 800-nt genomic tiles are indicated in the pie chart). Bottom: CpG methylation levels of meth− and escapees (meth+) in three species. White dots indicate the median, and black bars indicate the interquartile range. (F) Distribution of TE families that overlap with TE-rich escapees in week 5 pPGCs. Enrichment scores (ESs) of more than 2 for all Tes are shown, except for those marked by am asterisk, which had a score below 1. An ES above 2 and p < 0.001 (determined by Fisher’s test) indicates that the TE family is more frequent than what would be expected by chance. (G) Examples of TE-rich escapee loci overlapping with L1_SS, L1-2_SSc, and Pre0_SS. See also Figure S5 and Table S5.

We sought to obtain detailed information about pPGC DNA demethylation by generating whole-genome base-resolution PBAT libraries of week 5 (E35) gonadal pPGCs from 2 female and 2 male embryos (Table S1). In each replicate, over 90% of total genomic CpG sites were detected (i.e., covered with at least one read), and nearly 60% (apart from one sample of somatic cells [Soma.female], which is 52%) were covered by at least five reads (5×). The bisulfite conversion rate was around 99%, as determined with the spiked unmethylated lambda DNA (Table S5). Consistent with the LC-MS results (Figure 2B), week 5 pPGCs reached basal levels of DNA methylation (around 1%) in both genders, whereas gonadal somatic cells showed a median level of over 75% methylation (Figure 5A). WGBS-seq cannot discriminate between 5hmC and 5mC; however, based on the low levels of 5hmC determined by LC/MS-MS and IF, only a small proportion of the methylated DNA is likely to be enriched for 5hmC. It is not clear whether the low level of DNA methylation measured reflects resistance to demethylation or a low level of *de novo* methylation targeted at these regions.

Extensive DNA demethylation was determined across all genomic features, including CpG islands (CGIs), promoters, introns, intergenic regions, and exons (Figures 5B and 5C). Furthermore, week 5 PGCs also showed comprehensive demethylation of imprinted genes (Figure 5D), except for *PEG10*, which retained some methylation (7%–15%). The loss of DNA methylation at most imprinted loci in early gonadal germ cells is in line with previous reports showing that DNA demethylation at imprinted loci starts prior to arrival at the genital ridges (Hyldig et al., 2011a; Petkov et al., 2009).

Analysis of transposable elements (TEs), which are demethylated extensively in gonadal mPGCs and hPGCs (Hajkova et al., 2002; Seisenberger et al., 2012; Tang et al., 2015), also showed very low levels of DNA methylation in male and female pPGCs (Figure S5A), consistent with previous locus-specific analyses (Hyldig et al., 2011a; Petkov et al., 2009). DNA demethylation was concurrent with increased expression of major TE families, including long and short interspersed elements (LINEs and SINEs, respectively) and long terminal repeats (LTRs) in E14 and E31 (week 5) pPGCs (Figure S5B), in line with reports in gonadal hPGCs and mPGCs (Guo et al., 2015; Ohno et al., 2013; Hill et al., 2018). Concomitant upregulation of negative regulators of TE activity in PGCs, including *HELLS* and the piRNA pathway, suggests that mobilization of retrotransposons is likely to be repressed despite an increase in expression of TEs (Figure S2A; Table S6).

The overall low-level DNA methylation in week 5 pPGCs (∼1%) was comparable with that of week 7–9 hPGCs (∼4.5%) and E13.5 mPGCs (2.5%) (Kobayashi et al., 2013; Tang et al., 2015). Despite comprehensive demethylation, a small proportion of loci still maintained partial methylation (Figure 5E), as in the mouse and human (Guibert et al., 2012; Seisenberger et al., 2012; Tang et al., 2015). A large proportion of these loci are found in TE-abundant regions, whose distribution in the genome is variable, influencing the overall methylation levels in each species (Figures S5C and S5D). In the pig, the relative content of TEs (∼40%) in the genome is lower than in other mammals (Fang et al., 2012; Groenen et al., 2012), which could explain the reduced number of demethylation-resistant loci identified in this species (Figure 5E). We designated high-confidence demethylation-resistant loci escapees. Pig and mouse escapee loci are shorter than human escapees (Figure S6A). Notably, the most abundant repeat families at TE-rich (≥10% overlap with TEs) escapees are species-specific and evolutionarily young TEs, including the pig SINE element Pre0_SS of the PRE1 family, human AluY, and mouse IAPEz repeats (Figures 5F and 5G; Figure S5E). The overall observations in pig germ cells regarding global DNA demethylation and resistant loci parallel those in human and mice.

### TE-poor escapees show overlapping features between species

**Figure 6:**
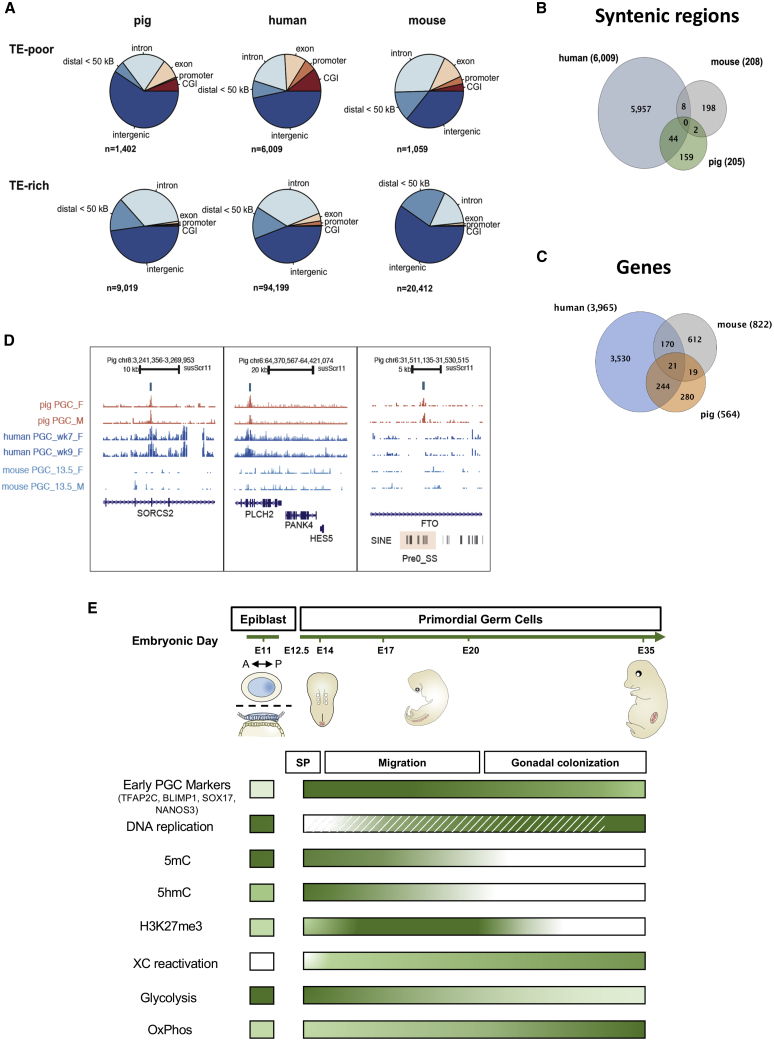
Common and unique features in DNA demethylation escapees between mouse, human, and pig (A) Distribution of TE-poor (<10% overlap with TEs) and TE-rich (≥10% overlap) escapees in week 5 pPGCs, week 7–9 hPGCs, and E13.5 mPGCs. The number of escapees (n) was determined by methylation level (at least 30% in human and 15% in pig and mouse). (B) Overlap of syntenic TE-poor escapees among pig, human, and mouse. Escapee regions in pig (205) and mouse (208) were lifted over to compare with syntenic regions in the human genome. (C) Overlap of homologous TE-poor escapee genes among pig, human, and mouse. (D) The TE-poor escapee regions within SORCS2 and PLCH2 are conserved between human and pig, whereas a pig-specific escapee is identified within FTO. (E) Diagram of the events in the pig germline. SP, germline specification; OxPhos, oxidative phosphorylation. A dashed line indicates expected DNA synthesis. See also Figure S6.

Many pig escapees at TE-poor (<10% overlap with TEs) regions are associated with promoters, CGIs, and gene bodies, as in hPGCs and mPGCs (Kobayashi et al., 2013; Tang et al., 2015). Their numbers vary, with the lowest in mPGCs (1,059) compared with pPGCs (1,402) and hPGCs (6,009) (Figure 6A). The larger proportion of TE-poor escapees (13%, 1,402 of 10,421) in pPGCs could be due to the relatively lower content of repetitive elements in the pig genome (Fang et al., 2012; Groenen et al., 2012; Figure 6A). Nearly 21.5% (44 of 205) of TE-poor escapee regions in the pig show conserved synteny with humans compared with 4% (8 of 206) in the mouse (Figure 6B). In addition, we found that 265 (47%) TE-poor escapee genes in the pig and 191 (23.2%) in the mouse are in common with human escapee genes (Figure 6C; Tang et al., 2015). Comparison with the NHGRI genome-wide association study (GWAS) catalog revealed that the 265 human-pig conserved TE-poor escapee genes are linked to metabolic and neurological traits, such as obesity-linked disorders and schizophrenia (Figure S6B). Some of the disease-associated genes show sequence conservation between human and pig, such as the obesity-related gene *SORCS2* and schizophrenia-related *PLCH2* (Figure 6D). For pig specific TE-poor escapee genes, comparison with the GWAS catalog revealed pig-specific terms, such as association with asthma (Figure S6C). TE-rich escapee genes overlapping with pig-specific TEs (*Pre0_SS* and *L1_SS*) also show enrichment for development-, metabolism-, and neurology-related GO terms, such as *FTO*, an obesity-related gene (Figure 6D; Figure S6D).

Last, analysis of common TE-poor escapee genes across at least two species (pig, human, and mouse) revealed enrichment for brain-specific gene expression, consistent with their association with neurology-related traits. These common genes also showed enrichment for key protein domains in the KRAB-ZFP family, suggesting a conserved mechanism for maintenance of methylation at these loci across species (Figure S6E).

### Conclusions

Our investigation advances insight into the mechanism of pPGC specification and their subsequent development. Notably, pPGC specification is closely linked to initiation of the epigenetic program in the absence of DNA replication, a unique germline property not seen in neighboring somatic cells (Figure 6E). There is a likely contribution through active mechanisms of DNA demethylation, as suggested by the conversion of 5mC to 5hmC, as well as upregulation of factors of the BER mechanism. Other factors associated with DNA repair are detected in early pPGCs at the time of epigenetic reprogramming, which is crucial for the germline that transmits genetic information to subsequent generations. The erasure of 5mC would necessitate alternative host defense mechanisms for the repression of TEs. Passive loss of 5mC during pPGC migration is also predicated because UHRF1 is repressed in early PGCs, a crucial factor for 5mC maintenance. Detection of several cell surface markers and transcriptional changes provide a basis to unravel how migration and subsequent development of pPGCs are regulated.

Observations on the human germline using *in vitro* models and *ex vivo* hPGCs (usually after week 5) concur with the events we observed in the early pig germline. Indeed, the initial studies of the critical factors and the mechanism of hPGC specification from *in vitro* models were confirmed by direct observations of pPGC specification in gastrulating pig embryos, suggesting that studies of the two species will be mutually informative. Importantly, investigations of very early hPGCs are exceptional (Tyser et al., 2020), especially during the critical period of weeks 2–4 of human development, when they are essentially inaccessible. Our observations of pPGCs over this critical period, covering specification and initiation of epigenetic reprogramming, likely apply to hPGCs.

Our study establishes a foundation for further investigations of the pig germline that will increase comprehension of the underlying developmental mechanisms. Porcine embryos are relatively accessible and ethically less challenging for studies. Genetic and other experimental approaches are possible with porcine embryos, which will lead to conceptual advances that will guide specific approaches for investigations of the human germline, including *in vitro* gametogenesis.

## STAR★methods

### KEY RESOURCES TABLE

**Table:** 

REAGENT or RESOURCE	SOURCE	IDENTIFIER
**Antibodies**		

Please see Table S7		N/A

**Biological samples**		

Pig embryonic tissues	Nottingham University Animal Unit	N/A
Human embryonic tissues	Addenbrooke’s Hospital, Cambridge, UK	N/A
Human ESCs	NHS Research Ethical Committee, UK	REC Number: 96/085

**Critical commercial assays**		

Nextera XT DNA Library Preparation Kit	Illumina	FC-131-1096
NEBNext Library Quant Kit	New England BioLabs	E7630L
Methylcode Bisulfite Conversion Kit	Invitrogen	MECOV-50
High Sensitivity DNA Kit	Agilent	5067-4626

**Deposited data**		

scRNA-seq and PBAT	This Paper	GEO accession: GSE155136

**Oligonucleotides**		

scRNA-seq oligonucleotides used, see Table S8	Picelli et al., 2014	N/A
PBAT oligonucleotides used in the study, see Table S8	Clark et al., 2017	N/A
Primers used for sexing pig embryos, see Table S8	Sembon et al., 2008	N/A
Primers used for sexing human embryos, see Table S8	Bryja and Konecny, 2003	N/A

**Software and algorithms**		

scythe (v0.981)	https://github.com/ucdavis-bioinformatics/scythe	https://github.com/ucdavis-bioinformatics/scythe
sickle (v1.33)	Joshi and Fass, 2011	https://github.com/najoshi/sickle
hisat2 (v2.1.0)	Kim et al., 2015	http://daehwankimlab.github.io/hisat2/
scater	McCarthy et al., 2017	http://bioconductor.org/packages/release/bioc/html/scater.html
SCDE	Kharchenko et al., 2014	https://hms-dbmi.github.io/scde/diffexp.html
picard (v2.12.1)	https://github.com/broadinstitute/picard	http://broadinstitute.github.io/picard/
GATK (v3.8)	Van der Auwera et al., 2013	https://github.com/broadinstitute/gatk/releases
Monocle 2 (v2.12.0)	Qiu et al., 2017	N/A
SnpEff (v4.3)	Cingolani et al., 2012	http://snpeff.sourceforge.net/
Seurat (v 3.1.2)	Stuart et al., 2019	https://cran.r-project.org/web/packages/Seurat/index.html
FastQC	Andrews, 2010	https://www.bioinformatics.babraham.ac.uk/projects/download.html#fastqc
Bismark	Krueger and Andrews, 2011	https://www.bioinformatics.babraham.ac.uk/projects/download.html#bismark
MethPipe	Song et al., 2013	http://smithlabresearch.org/software/methpipe/
featureCounts	Liao et al., 2014	https://bioconductor.org/packages/release/bioc/html/Rsubread.html
Fiji	Schindelin et al., 2012	https://imagej.net/Fiji#Downloads

**Other**		

Illumina Hiseq 2500	Illumina	N/A
Illumina Hiseq 4000	Illumina	N/A
Fluorescence Microscope DMIR	Leica	N/A
UHPLC 1290 System	Agilent	N/A
6490 Triple Quadrupole mass spectrometer	Agilent	N/A
MoFlo XDP Cell Sorter	Beckman Coulter	N/A
S3 Cell Sorter	Bio-Rad	N/A
SH800Z Cell Sorter	Sony	N/A
Agilent 2100 Bioanalyzer	Agilent	N/A

### Resource availability

#### Lead contact

Further information and requests for resources and reagents should be directed to and will be fulfilled by lead contact Ramiro Alberio (ramiro.alberio@nottingham.ac.uk).

#### Materials availability

This study did not generate new unique reagents.

#### Data and code availability

The scRNaseq and PBAT data generated under this study can be accessed from GEO: GSE155136.

### Experimental model and subject details

#### Pig embryos and PGCs collection

All the procedures involving animals have been approved by the School of Biosciences Ethics Review Committee, The University of Nottingham. Embryos were retrieved from crossbred Large White and Landrace sows (2–3 years old) between days 11 to 35 after artificial insemination. E11 and E14 embryos were flushed from the uterine horns with warm washing buffer (PBS supplemented with 1% fetal bovine serum (FBS)). Later stage embryos (> E25) were manually dissected from the uterine horns and washed with washing buffer. Epiblast from E11 embryos were manually dissected and stored at −80°C before further processing for LC-MS (see below). PCR was used for sex identification of E35 embryos before processed for FACS and PBAT library preparation (Sembon et al., 2008; Table S8).

Pig PGC isolation was carried out as previously described (Hyldig et al., 2011a). Briefly, embryos between E14 to E35 were stored in DMEM/F-12 supplemented with 40% FBS at 4°C overnight before being processed the next day. Dissected posterior ends of E14 embryos containing PGC clusters and gonads from E31 and E35 embryos were digested at 37 °C for 30 mins using Collagenase IV (2mg/ml in DMEM), with gentle pipetting every 5 mins. The cell suspension was washed with DMEM, centrifuged and the pellet re-suspended in TrypLE Express (GIBCO) for further digestion at 37°C for 3-5 mins. Enzymatic digestion was neutralized with dissection medium (DMEM/F-12 with 10% FBS, 25 mM HEPES and 100 U/ml Penicillin-0.1 mg/ml Streptomycin). The cell suspension was filtered through a 40 μm cell strainer into FACS tube. Following centrifugation, cells were re-suspended and incubated in dissection medium with Sda/GM2 antibody (Klisch et al., 2011) for 30 mins at 4°C. After washing with dissection medium, cells were re-suspended and incubated in dissection medium with Alexa 488 Donkey Anti-Mouse for 30 mins, and then diluted with dissection media and FACS sorted by MoFlo XDP. For PBAT, E35 Sda/GM2+ cells were sorted twice to ensure high purity.

#### Human embryonic tissues and collection of hPGCs

Human embryonic tissues were used under permission from NHS Research Ethical Committee, UK (REC Number: 96/085). Human embryonic samples were collected following medical or surgical termination of pregnancy carried out at Addenbrooke’s Hospital, Cambridge, UK with full consent from patients. Crown-rump length, anatomical features, including limb and digit development, was used to determine developmental stage of human embryos with reference to Carnegie staging (CS). The sex of embryos was determined by sex determination PCR, as previously described (Bryja and Konecny, 2003).

Human embryonic genital ridges from two individual male embryos (developmental week 7-8, Carnegie stage 19) were dissected in PBS and separated from surrounding mesonephric tissues. The embryonic tissues were dissociated with 100 μl TrypLE Express (Life Technologies) at 37°C for 30 minutes. Tissues were pipette up and down for ten times every 5 minutes to facilitate dissociation into single cell suspension. After that, samples were diluted with 100 μl FACS medium (PBS with 3% FBS & 5 mM EDTA) and centrifuged at 500 xg for 5 minutes. Cell pellet was suspended with FACS medium and incubated with 5 μl of Alexa Fluor 488-conjugated anti-alkaline phosphatase (AP) (BD PharMingen, 561495) and 25 μl of PerCP-Cy5.5-conjugated anti-CD117 (BD PharMingen 333950) antibodies for 15 minutes at room temperature with rotation at 10 revolutions per minutes (rpm) in dark. Cell suspension was then diluted in 1 mL FACS medium and centrifuged at 500 xg for 5 minutes. After removing the supernatant, the cell pellet was resuspended in FACS medium and passed through a 35μm cell strainer. Samples were subjected to FACS using the S3 Cell Sorter (Bio-Rad). hPGCs (AP- and CD117-positive) and the neighboring gonadal somatic cells (AP- and CD117-negative) were collected and stored at −80°C until mass spectrometry analysis.

#### Human ESC culture, hPGCLC induction and collection

Male hESCs with a NANOS3–tdTomato reporter was established previously (Kobayashi et al., 2017) and confirmed as mycoplasma negative. hESCs were maintained on vitronectin-coated plates in Essential 8 medium (Thermo Fisher Scientific) according to manufacturer’s protocol. Cells were passed every 3-5 days using 0.5 mM EDTA in PBS without breaking cell clumps.

hPGCLCs were generated using a two-step protocol as described before (Kobayashi et al., 2017). Briefly, trypsinized hESCs were seeded on vitronectin-coated dish at 200,000 cells per well in 12-well plate and cultured in mesendoderm induction medium for 12 hours. Mesendoderm medium consisted of aRB27 basal medium (Advanced RPMI 1640 Medium (Thermo Fisher Scientific) supplemented with 1% B27 supplement (Thermo Fisher Scientific), 0.1 mM NEAA, 100 U/ml penicillin, 0.1 mg/ml streptomycin, 2 mM L-glutamine), 100 ng/ml activin A (Department of Biochemistry, University of Cambridge), 3 μM GSK3i (Miltenyi Biotec) and 10 μM of ROCKi (Y-27632, Tocris Bioscience).

To induce hPGCLCs, pre-mesendoderm cells were trypsinized into single cells and harvested into Corning Costar Ultra-Low attachment multiwell 96-well plate (Sigma) at 4,000 cells per well in hPGCLC induction medium, which composed of aRB27 medium supplemented with 500 ng/ml BMP4,10 ng/ml human LIF (Department of Biochemistry), 100 ng/ml SCF (R&D systems), 50 ng/ml EGF (R&D Systems), 10 μM ROCKi, and 0.25% (v/v) poly-vinyl alcohol (Sigma). Cells were cultured as floating aggregate for 5 days. Aggregates were trypsinized with 0.25% trypsin/EDTA at 37°C for 5-15 min. Cell suspension was subjected to FACS by SH800Z Cell Sorter (Sony). NANOS3–tdTomato-positive hPGCLCs and NANOS3–tdTomato-negative neighboring cells were collected for mass spectrometry analysis.

### Method details

#### Isolation of single cells for single-cell library preparation

FACS sorted cells were washed in a small drop of PBS-PVP and single cells were manually collected with thin capillaries and placed into PCR tubes to prepare single-cell cDNA libraries following the Smart-seq2 protocol (Picelli et al., 2014).

Briefly, single cells were lysed by incubation at 72 °C for 3 min in PCR tubes containing 4 μl of cell lysis buffer, oligo-dT primer and dNTP mix. Reverse transcription and PCR pre-amplification were carried out with SuperScript II (Invitrogen) and KAPA HiFi HotStart ReadyMix (KAPA Biosystems) respectively according to Picelli et al. (2014). PCR products were purified using Ampure XP beads (Beckman Coulter), and library size distribution was checked on Agilent dsDNA High Sensitivity DNA chips on an Agilent 2100 Bioanalyzer (Agilent Technologies). Concentration was quantified using Qubit Quant-iT dsDNA High-Sensitivity Assay Kit (Invitrogen). Samples with more than 0.2 ng μl^−1^, free of short fragments (< 500 bp) and with a peak at around 1.5–2 kb were selected for library preparation with Nextera XT DNA Library Preparation Kit (Illumina). Tagmentation reaction and further PCR amplification for 12 cycles were carried out, and PCR products were again purified using Ampure XP beads. Quality of the final cDNA library was analyzed on an Agilent high sensitivity DNA chip. Final cDNA libraries had an average size of 700–800 bp and were quantified using NEBNext Library Quant Kit for Illumina (New England BioLabs) following the manufacturer instructions. Finally, libraries were pooled in groups of 50 with a 2 nM final concentration, and DNA sequencing was performed on a HiSeq 2500 Sequencing System (Illumina). Oligonucleotides used as described in Table S8.

#### Single-cell RNA-Seq data analysis

Raw PE reads were trimmed against adaptor sequences by scythe (v0.981), and quality-trimmed by sickle (v1.33) using default settings. Trimmed reads were directionally aligned to the pig genome (Sus scrofa v11) by hisat2 (v2.1.0) with *-know-splicestie-infile* setting to increase mapping accuracy of splicing reads. Uniquely and correctly mapped reads were extracted for the downstream analysis. htseq-count was used to count the number of reads aligned to each gene (Sus scrofa v11.2 ensembl annotation build 91). Gene expression level was calculated and normalized by Transcripts Per Kilobase Million (TPM).

Low quality cells were filtered out from the dataset to reduce the downstream analysis noise. First, the total number of reads mapped to gene transcripts was calculated for each cell, and those with less than 1 million were removed. Second, the proportion of reads aligned to mitochondrial genes was estimated, as a high proportion suggests poor quality cells (Ilicic et al., 2016). The proportion cut-off was set at 0.5. Only cells of proportions below 0.5 were kept for the next analysis. Third, 2 outlier cells were identified by t-SNE dimensionality reduction. A total of 14,873 out of 25,880 annotated genes were identified in at least 3 cells with TPM > 1.

The R package “scater” was applied to normalize read counts of genes for each good quality cell with acceptable sequencing coverage. A non-linear approach, t-stochastic neighbor embedding (t-SNE), was used to identify the relations between cells using normalized read counts. Unsupervised hierarchical clustering using all expressed genes as input was conducted on all filtered cells by normalized read counts in log2 scale. The distance method was euclidean, and the cluster method was ward.D2.

#### Differential expression and enrichment analysis

Pairwise comparisons of single-cell differential expressions were performed by SCDE using normalized read counts among four embryo stages. Two-tailed adjusted p value were calculated using cZ scores from Benjamini–Hochberg multiple testing corrections, which followed a normal distribution. Significantly expressed genes were selected with a p value < 0.05 as the threshold. Euclidean distance and default hclust were applied to determine the relationships between cells and between genes. Gene Ontology (GO) gene set enrichment analysis with DEGs utilized goseq for each pairwise comparison, also with upregulated DEGs and downregulated DEGs separately. GO term annotation was retrieved from the Ensembl database (Sus scrofa v11.1 ensembl annotation version 91). Enrichment analysis of biological pathways (KEGG) was performed with DEGs by R package “clusterProfiler.” Ensembl gene IDs of DEGs were mapped to NCBI gene IDs for KEGG pathway prior to enrichment analysis.

#### Inference of embryonic sex

Expressions of all the single-copy genes on chrY were summed up to determine the gender of each cell. First, any cell with the total TPM of chrY single-copy genes ≥ 10 was regarded a male cell. Others were regarded as female cells. Then, the ratios of the total gene expressions between chrY and chrX (∑ ChrY Total TPM / ∑ ChrX Total TPM) were calculated across all cells. Any pre-determined male cell with the ratio lower than the maximal ratio of pre-determined female cells was regarded as the female cell.

#### Chromosome X dosage compensation analysis

Genes of chromosome X and three autosomes (chr1, chr2, chr3) were extracted, and the geometric mean TPM of chromosomal expressed genes was calculated for each cell separately. Then the overall geometric mean TPM was obtained for each developmental stage by embryo sex, as well as the total TPM. Each TPM value was incremental by one (TPM + 1) for the calculation of geometric mean TPM. Only shared expressed genes between female and male cells were taken into account in the calculation of female/male expression ratio for each chromosome. Median Female/Male expression ratio was estimated for each stage across the whole chromosome X with 1 Mb window. The ratio of chrX/auto in each cell was inferred by the median value of bootstrapped ratios. Each ratio was estimated by the total TPMs of a certain number of random-selected genes. The median ratios were grouped by embryo sex.

#### Analyses of allelic expression

Trimmed reads were aligned to chromosome X of the pig genome (Sus Scrofa v11.1) by hisat2. Duplicated reads were marked by picard (v2.12.1). GATK (v3.8) was used to retrieve allelic read counts for SNVs annotated in dbSNP. Only validated SNVs (dbSNP flag VLD) were extracted for downstream analysis. SnpEff (v4.3) was applied to annotate called SNVs with Sus scrofa v11.1 ensembl annotation. Low coverage SNVs (< 3 reads) were excluded from the analysis, and we only kept SNVs that occurred at least in two different cells for each stage. The expressions of mono-/bi-allelic genes were inferred based on SNVs in each female cell of each stage.

#### Single cell trajectory analysis

Trajectory modeling and pseudotemporal ordering of cells was performed using TPM data with Monocle 2 (Qiu et al., 2017) (version 2.12.0). Top 1000 significant differentially expressed genes between clusters were used for ordering the cells.

#### Comparison of pig, human and cynomolgus monkey datasets

In total, dataset of E14-31 pig cells (128 from our study), processed data of Wk4-7 human cells (149) retrieved from GSE86146 (Li et al., 2017) and processed data of E13-55 cy monkey cells (100) retrieved from GEO: GSE76267, GSE74767 and GSE67259 (Sasaki et al., 2016) were included in the comparison. Natural log-transformed, pre-normalized expression matrix of common genes (i.e., homologs genes with same gene name) across three species were imported and processed by *FindIntegrationAnchors* and *IntegrateData* (k.filters set as “NA”) functions in Seurat (version 3.1.2) (Stuart et al., 2019). Dimensionality reduction by *RunUMAP* with default settings was then performed for the integrated dataset.

Expression of selected lineage markers, membrane proteins and epigenetic modifiers in E14-31 pig cells, CS7 human cells, Wk4-7 human cells and E13-55 *Cynomolgus* cells were plotted separately with pheatmap package (Kolde, 2015).

#### Cell cycle analysis

Default settings of *CellCycleScoring* function in Seurat were used to score the cell cycle phases of each single cell. In brief, single cells were assigned a score with *AddModuleScore* function based on its expression of G2/M- and S-phase markers provided in Seurat. The single cells highly expressing G2/M- or S-phase markers were assigned as G2/M- or S-phase cells, respectively, and the single cells not expressing any of the two categories of genes were assigned as G1 phase.

#### Signature set analysis

With the processed single cell RNA-seq data of pig embryos from Ramos-Ibeas et al. (2019) we used *FindMarkers* function in Seurat (Wilcoxon rank sum test) to identify the highly expressed genes (avg_logFC > = 1 and adjusted.p ≤ 0.05) as the signature set in E6 ICM, E8 epiblast and E11 epiblast. Next, we calculated the relative average expression level of each signature set with *AddModuleScore* function of Seurat in single cells of E14 Soma, E14 PGC and E31 PGC, which was then visualized by heatmap using pheatmap package.

#### PBAT library construction

PBAT libraries were prepared as described previously (Tang et al., 2015) with some modifications. The Sda/GM2-positive (PGCs) and -negative (Somatic) cells collected by FACS were lysed with lysis buffer (0.1% SDS, 50 ng/ml carrier RNA (QIAGEN) and 1 mg/ml proteinase K (Zymo Research) in DNase-free water) for 60 min at 37°C. Unmethylated lambda phage DNA (0.2 ng/sample) (Promega) was spiked into the sample before bisulfite treatment with the Methylcode Bisulfite Conversion Kit (Invitrogen) according to the manufacturer’s instructions, except that the bisulfite conversion step was increased to 3.5 hours. Bisulfite-treated DNA was re-annealed to double-stranded DNA using Klenow fragments (3′–5′ exo-) (New England Biolabs) with a 5′ biotin tagged primer consisted of an Illumina adaptor followed by 6 random nucleotides (Clark et al., 2017; Table S8).

The biotinylated first strand molecules were captured using Dynabeads M280 Streptavidin (Invitrogen) and then reannealed to double-stranded DNA again using Klenow fragments (3′–5′ exo-) with random primers containing Illumina adaptors (Clark et al., 2017).

Template DNA strands were then synthesized as cDNA with a second strand (where unmethylated C’s were converted to T’s) and then amplified with 11 cycles using KAPA HiFi HotStart Readymix (Roche) with the Illumina primer and iPCRTag

Size fractionation was performed on the eluted DNA with Agencourt AMPure XP (Beckman Coulter). Concentrations of PBAT libraries were determined by qPCR using NEBNext Library Quant kit (NEB). Libraries were subjected to paired-read 150bp sequencing on HiSeq 4000 sequencing system (Illumina). Coverage information was summarized in Table S5.

#### DNA methylation analysis

The quality of raw reads was determined by FastQC to ensure that the experimental setup and sequencing were successful. Raw reads were trimmed by skewer first to remove adaptor sequences and reads with low sequencing qualities (Jiang et al., 2014). Then, both the ends of paired-end reads were trimmed to improve the mapping efficiency. Forward reads were trimmed by 10 bases at the beginning, while reverse reads were trimmed by 5 bases at the end.

Trimmed reads were directionally aligned against the pig genome (Sus Scrofa v11.1) in the paired-end mode by hisat2 using Bismark pipeline with *–pbat*.–score_min was L,0,-0.4. *deduplicate_bismark* was applied to remove the potential PCR duplicates with default settings (Krueger and Andrews, 2011). Unmapped reads were re-aligned with the same parameters in the single-end non-direction mode to rescue misaligned paired-end reads due to the incorrect insert size resulting from the narrow sequencing area. The single-end alignment was merged with the paired-end alignment after deduplication.

To compare the pig PBAT datasets with those from human (Tang et al., 2015) and mouse (Kobayashi et al., 2013), reads were trimmed up to 100 nt for all three species, and were mapped via single-end only and sampled to the same depth.

The detection of methylated cytosines was done by *bismark_methylation_extractor*, which can provide the genome-wide cytosine methylation status. The spike-in unmethylated lambda phage DNA was also included in the analysis to examine the efficiency of bisulphite conversion in the samples.

The annotation of the methylation level was calculated by the module *roimethstat* of *MethPipe* according to the locations of CpG islands and CGI shores, the genomic features and by the repeat density (Song et al., 2013). Annotations of CpG islands, genes, promoters and repeat regions were downloaded from UCSC and Ensembl databases. Promoter regions were defined as sequences located between 1,000 bp upstream and 500 bp downstream of a transcription start site. Promoters with high-CpG content (HCP) contain a 500 bp region with a CpG ratio larger than 0.75 and a GC content larger than 55%. Promoters with low-CpG content (LCP) do not contain a 500-bp region with a CpG ratio larger than 0.48. Intermediate-CpG promoters (ICPs) are neither HCP nor LCP.

Hypermethylated regions (HyperMR) were identified by the *hmr* function of *MethPipe*. Escapees were defined as regions which have more than 20% of CpGs with > = 5x with at least 30% methylation level in human and 15% in pig and mouse. TE-poor escapees were defined as less than 10% of regions overlapped with repeats. TE-rich escapees were defined as more than 10% of regions overlapped with repeats.

#### TE Expression Analysis

Repeat regions were downloaded from UCSC database including all the sub families. *featureCounts* (Liao et al., 2014) was used to determine the number of reads aligned to each region with *-M* option. To avoid multiple mapping of reads we applied a cut-off for mapping quality score, which was set as 20 (i.e., -Q 20). Expression level was calculated and normalized by Reads Per Kilobase Million (RPM).

#### Immunofluorescence staining of porcine tissues

Embryos were processed as previously described (Kobayashi et al., 2017). Briefly, embryos and gonads were fixed in 4% paraformaldehyde (PFA)/PBS overnight (ON) at 4°C. Fixed embryos were incubated in 30% sucrose/PBS for two days at 4°C prior to mounting in optimal cutting temperature (OCT) compound. Cryosections were cut at 5-7 μm onto Superfrost plus glass slides. Sections were left to air dry for 1-2 h before IF.

For IF, cryosections were washed with PBS for 10 mins to remove OCT compound. Antigen retrieval was then performed by boiling the slides in 0.01M Citrate Buffer (pH 6.0) for 10 min. Sections were permeabilized with 1% Triton X-100 in PBS for 15 min. Triton X-100 was washed three times for 5 min each, and blocking solution (PBS supplemented with 5% BSA and 10% Donkey serum) was added for 1.5 h. After blocking, sections were incubated with the desired primary antibody (Table S7) ON at 4°C in a humidified chamber. Slides were then washed three times with 0.1% Tween-20/PBS. Slides were then incubated with fluorescent (Alexa Fluorophore 488, 555, and/or 647; Invitrogen)-conjugated secondary antibodies for 40 min at room temperature (RT). Slides were mounted with Fluoroshield with DAPI (Sigma) and sealed with nail varnish. Slides were kept at −20°C until observed.

Image acquisition was performed using SimplePCI capture software on an epifluorescence microscope (Leica). Fiji was used for cell count and fluorescence quantification of ROI (Schindelin et al., 2012). For fluorescence quantification, background intensity was subtracted to generate corrected total cell fluorescence (CTCF), i.e., CTCF = Integrated Density – (Area of selected cell X Mean fluorescence of background readings) (McCloy et al., 2014).

#### Mass spectrometry

Genomic DNA from E11 epiblast and FACS-sorted pPGCs was extracted using Quick-DNA/RNA Miniprep kit (Zymo Reasearch) following the manufacturer’s instructions and eluted in LC–MS grade water. DNA was digested to nucleosides using a using a nucleoside digestion mix (NEB). The nucleosides were separated on an RRHD Eclipse Plus C18 2.1 × 100 mm 1.8u column using the HPLC 1290 system (Agilent) and mobile phases 100% water 0.1% formic acids and 80% methanol, 0.1% formic acids. Quantification was carried out in an Agilent 6490 triple quadrupole mass spectrometer on multiple reaction monitoring mode (MRM). To calculate the concentrations of individual nucleosides, standard curves were generated (dC and dG from Berry and Associated; 5mdC and 5hmdC from CarboSynth). All samples and standard curve points were spiked with a similar amount of isotope-labeled synthetic nucleosides (13C15N-dC and 13C15N-dG purchased from Silantes, and d3-mdC and d215N2-mhdC was obtained from T. Carell (Center for Integrated Protein Science at the Department of Chemistry, Ludwig-Maximilians-Universität München, Germany). The threshold for quantification is a signal-to-noise above ten (calculated with a peak-to-peak method). Limit of quantification (LOQ) was 0.025 fmol for 5mdC and 5hmdC, and 0.5 fmol for dC and dG.

### Quantification and statistical analysis

Statistical differences in 5hmC and 5mC levels determined by LC–MS, were determined with ANOVA and Holm’s post hoc test. Differences in female to male expression ratio across X chromosome and X:A ratio in E14 PGC, E31 PGC and E14 Somatic cells, were calculated using pairwise Wilcoxon test. To evaluate the statistical differences in number of biallelically expressed genes in E14 PGC, E31 PGC and E14 Somatic cells, p value is determined by Kruskal-Wallis test followed by Dunn’s test. Statistical differences in *KDM6A* expression in E14 cells, *was calculated* using Mann-Whitney U-test. Differences in H3K27me3 quantification in E17 migratory PGC and surrounding somatic cells, were calculated with Mann-Whitney U-test. Differences in expression profiles of major TE families in E11 epiblast, E14 somatic cells, E14 and E31 PGCs were calculated using pairwise Wilcoxon test.

## Supplemental information

Document S1. Figures S1–S6 and Table S8

Table S1. List of samples used in this study, related to Figure 1

Table S2. GO terms and KEGG enrichment analysis of DEGs, related to Figure 1

Table S3. DEG comparison of pig and cynomolgus monkey, related to Figure 1

Table S4. Pluripotency signature gene sets, related to Figure S1

Table S5. Sequencing statistics for PBAT, related to Figure 5

Table S6. PGC-high TE repressors, related to Figure S2

Table S7. Antibody list, related to STAR methods

Document S2. Article plus supplemental information
