# Supplementary material for: Specification and epigenomic resetting of the pig germline exhibit conservation with the human lineage
Source: Cell Rep. 2021 Feb 9;34(6):108735. doi: 10.1016/j.celrep.2021.108735 (PMC7873836; doi:10.1016/j.celrep.2021.108735)
Supplement: Document S1. Figures S1–S6 and Table S8 [file mmc1.pdf]

Cell Reports, Volume *34*

Supplemental Information

Specification and epigenomic resetting of the pig germline exhibit conservation with the human lineage

Qifan Zhu, Fei Sang, Sarah Withey, Walfred Tang, Sabine Dietmann, Doris Klisch, Priscila Ramos-Ibeas, Haixin Zhang, Cristina E. Requena, Petra Hajkova, Matt Loose, M. Azim Surani, and Ramiro Alberio

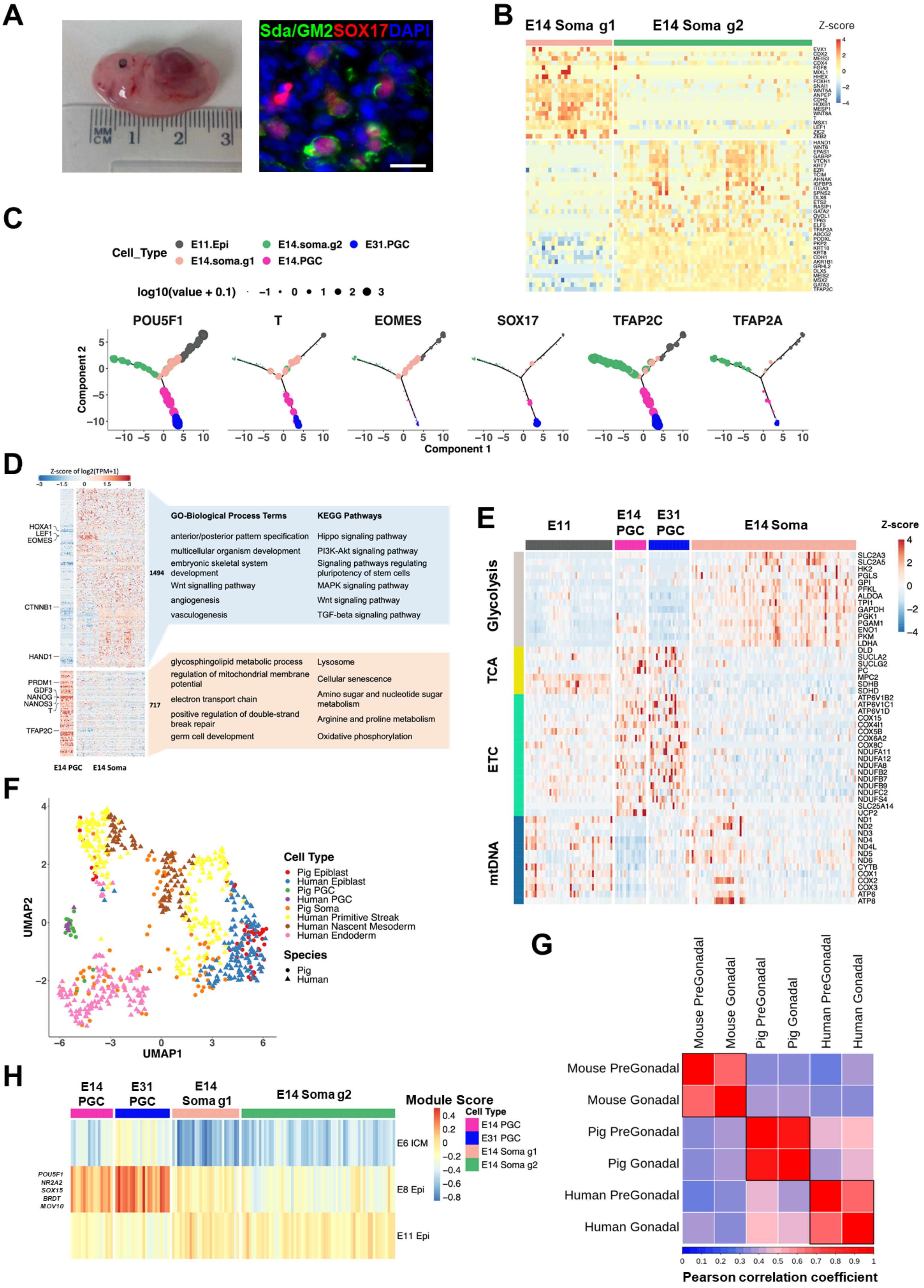

Figure S1. Gene expression differences between pPGC and surrounding cells. Related to Figure 1.
(A) Image of E31 foetus (left) and immunostaining of a section of E31 gonad showing expression of the cell surface marker Sda/GM2 and SOX17 in PGCs (right). Scale bar: 20μm.
(B) Expression heatmap of DEGs in somatic cells found in the posterior end of E14 embryos. Colour scale unit: Z-score of TPM.
(C) Pseudotime expression of selected lineage markers on single cell trajectories.
(D) Gene expression heatmap, GO terms and KEGG pathways for DEGs between E14 PGCs and somatic cells. See also Table S2. Colour scale unit: Z-score of log(TPM+1).
(E) Gene expression heatmap of cellular metabolism and mitochondrial DEGs in different cells types. ETC: electron transport chain. mtDNA: mtDNA-encoded components. TCA: tricarboxylic cycle. Colour scale unit: Z-score of TPM.
(F) UMAP plot showing integration of human CS7 cells (Tyser et al., 2020) and pig (E14) pPGCs and somatic cells.
(G) Heatmap representation of the correlation coefficient between human, mouse and pig PGCs.
(H) Relative average expression (module score) of signature sets of E6, E8 and E11 cells in E14 and E31 cells.

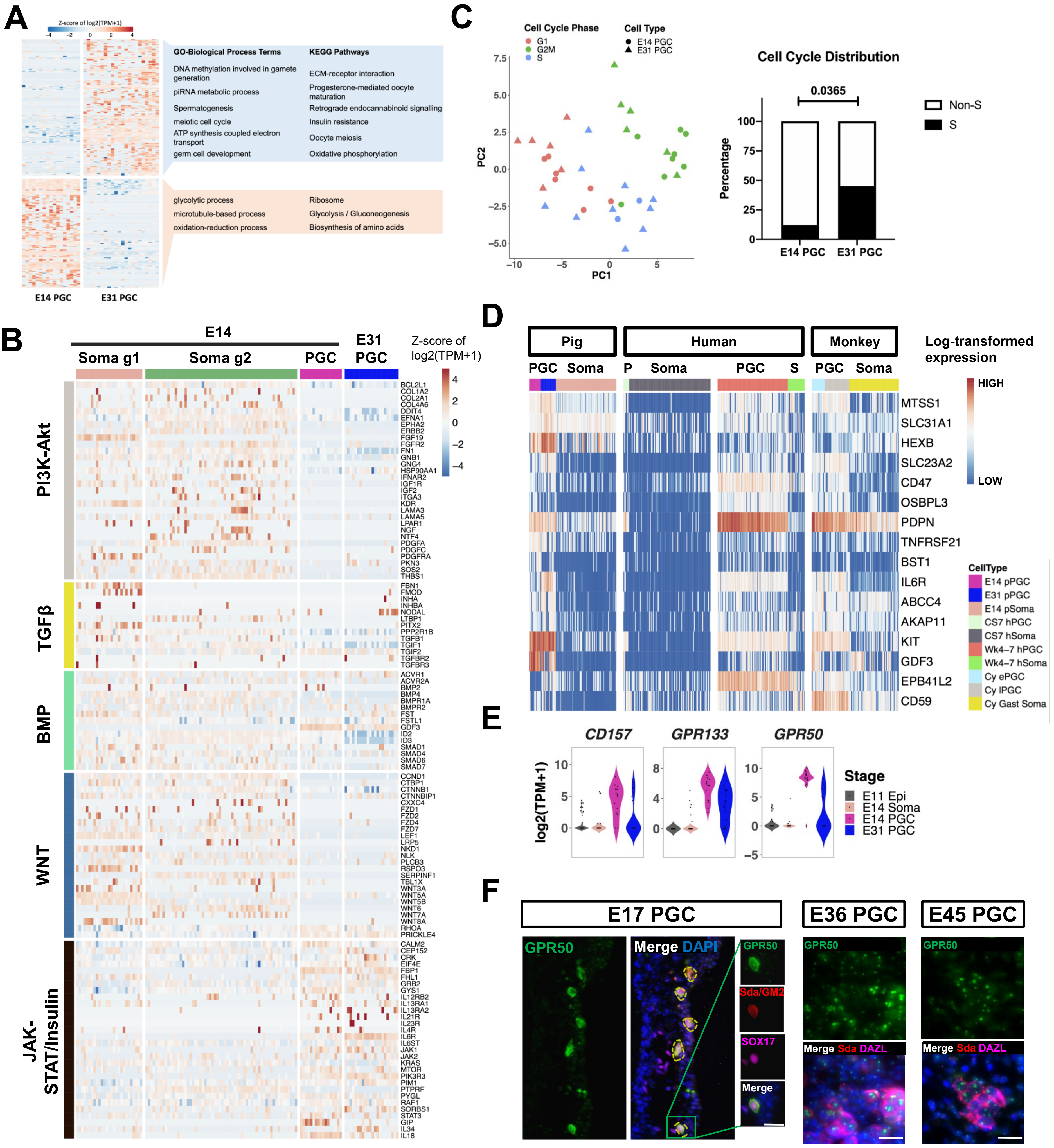

Figure S2. Molecular differences between E14 and E31 pPGC, Related to Figure 1.
(A) Gene expression heatmap, GO terms and KEGG pathways for DEGs between E14 and E31 PGCs. Colour scale unit: Z-score of log(TPM+1). See also Table S2.
(B) Heatmap of selected genes from different signalling pathways expressed in E14 and E31 cells. Colour scale unit: Z-score of log(TPM+1).
(C) Determination of cell cycle stage of E14 and E31 PGCs based on relative average expression (module score) of canonical cell cycle markers (left). Analysis of cell cycle stage distribution in E14 (G1+G2=15; S=2 cells) and E31 (G1+G2=12; S= 10 cells) analysed by Fisher's exact Test (right).
(D) Expression heatmap of cell surface and membrane proteins in hPGCs, cyPGCs and pPGCs compared to somatic cells. Wk (week) 4-7 (Li et al., 2017); Cy ePGC: early cyPGC (E13-20); Cy lPGC: late cyPGC (E36-55); Cy Gast Soma: cy gastrulating cells (E13-20) (Sasaki et al., 2016). CS7 hPGC (Carnegie stage 7) PGCs and soma (Tyser et al., 2020); S: Soma. P: PGCs. Z-score of log-transformed matrixes were used. As different expressional units are used for the three species, values in the colour scale are replaced by HIGH and LOW.
(E) Violin plots showing expression of cell surface proteins (CD157 (*BST1*), GPR133 (*ADGRD1*) and GPR50) in pPGCs compared to soma and Epi.
(F) GPR50 detected by IF in E17, E36 and E45 PGCs (indicated by yellow circles). PGCs are marked by SOX17 and Sda/GM2 in E17 and by DAZL in E36 and E45. Scale bar: 20µm.

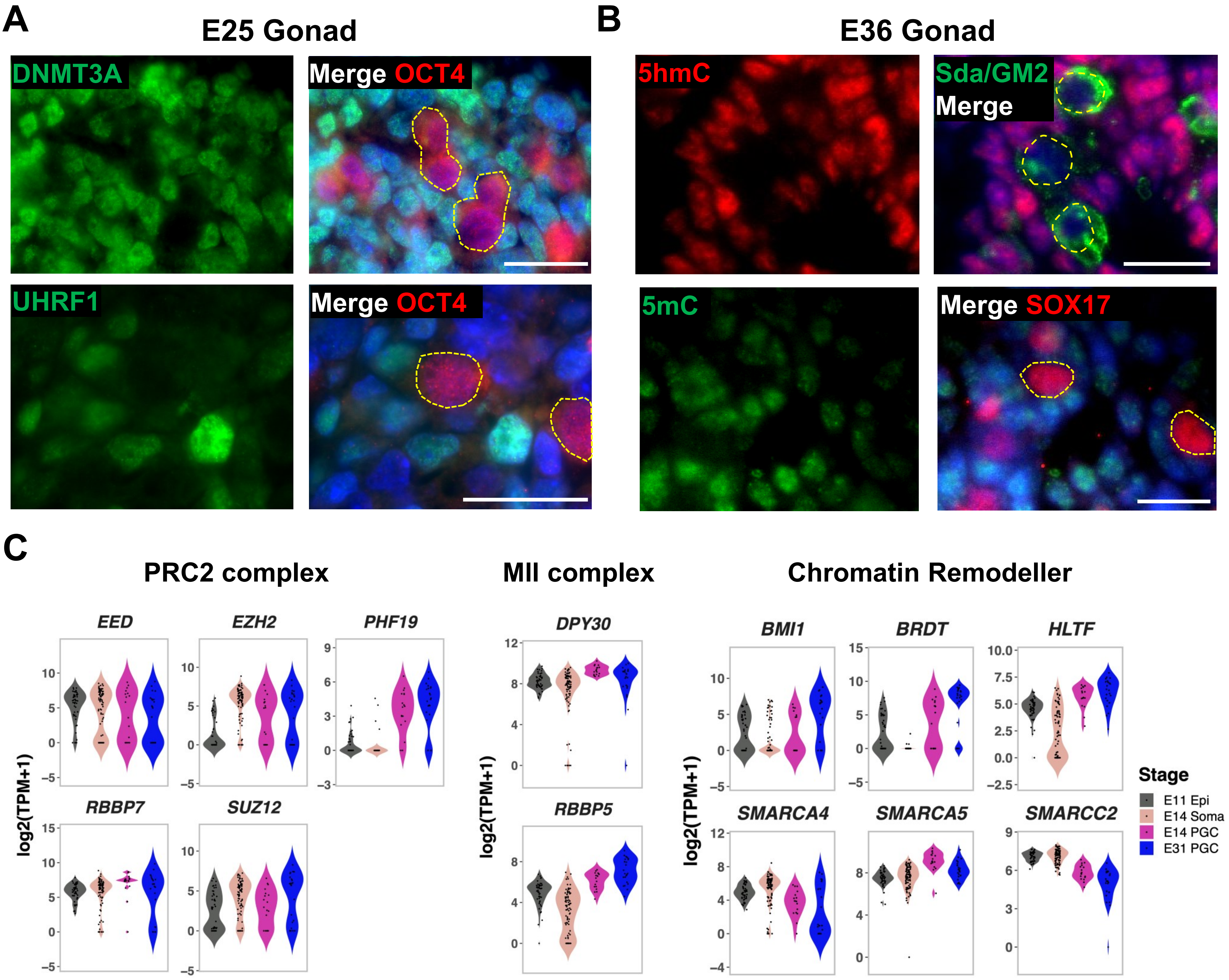

Figure S3. Epigenetic reprogramming in pPGCs. Related to Figure 2 and Figure 3.
(A) Expression of DNMT3A and UHRF1 in E25 Gonadal pPGCs determined by IF. PGCs are marked by OCT4 (red). Scale bar: 20μm. Yellow circles indicate PGC.
(B) Immunofluorescence of 5hmC (top) and 5mC(bottom) in E36 Gonadal PGCs. PGCs are marked by Sda/GM2 (top, green) and SOX17 (bottom, red). Scale bar: 20μm. Yellow circles indicate PGC.
(C) Expression profile of selected components of PRC2 complex, Mll complex and chromatin remodellers in E11 Epiblast cells, E14 Somatic cells, E14 PGCs and E31 PGCs.

A

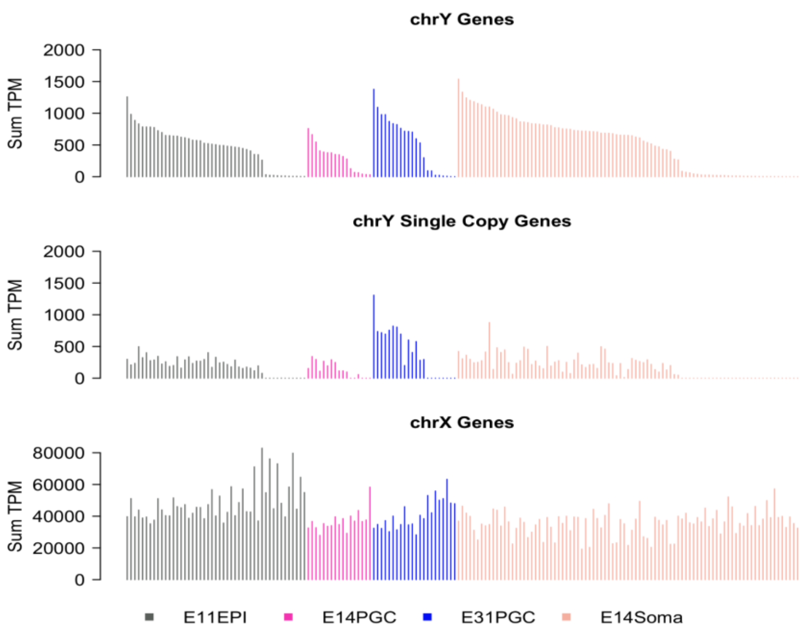

B

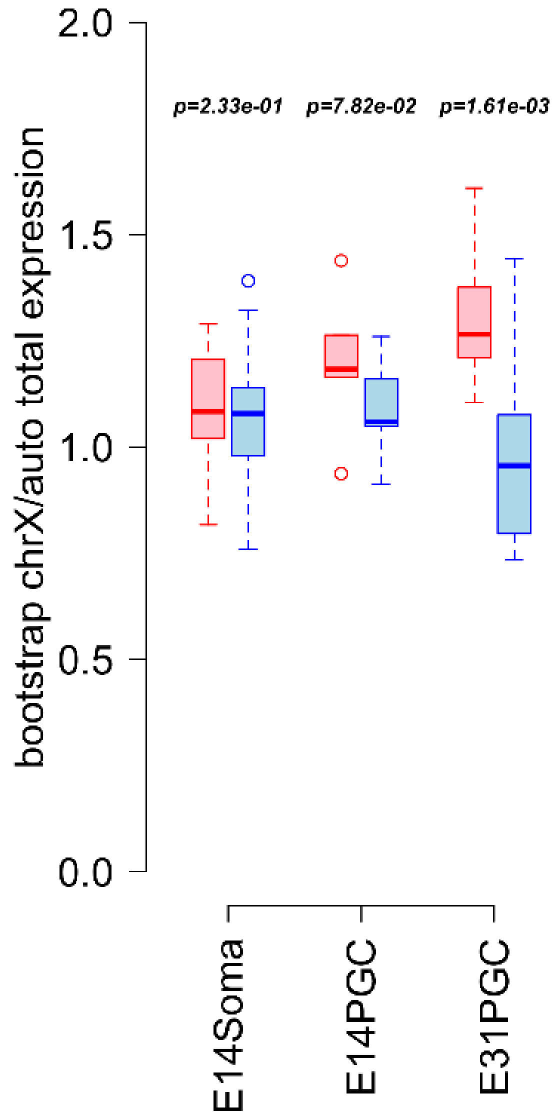

C

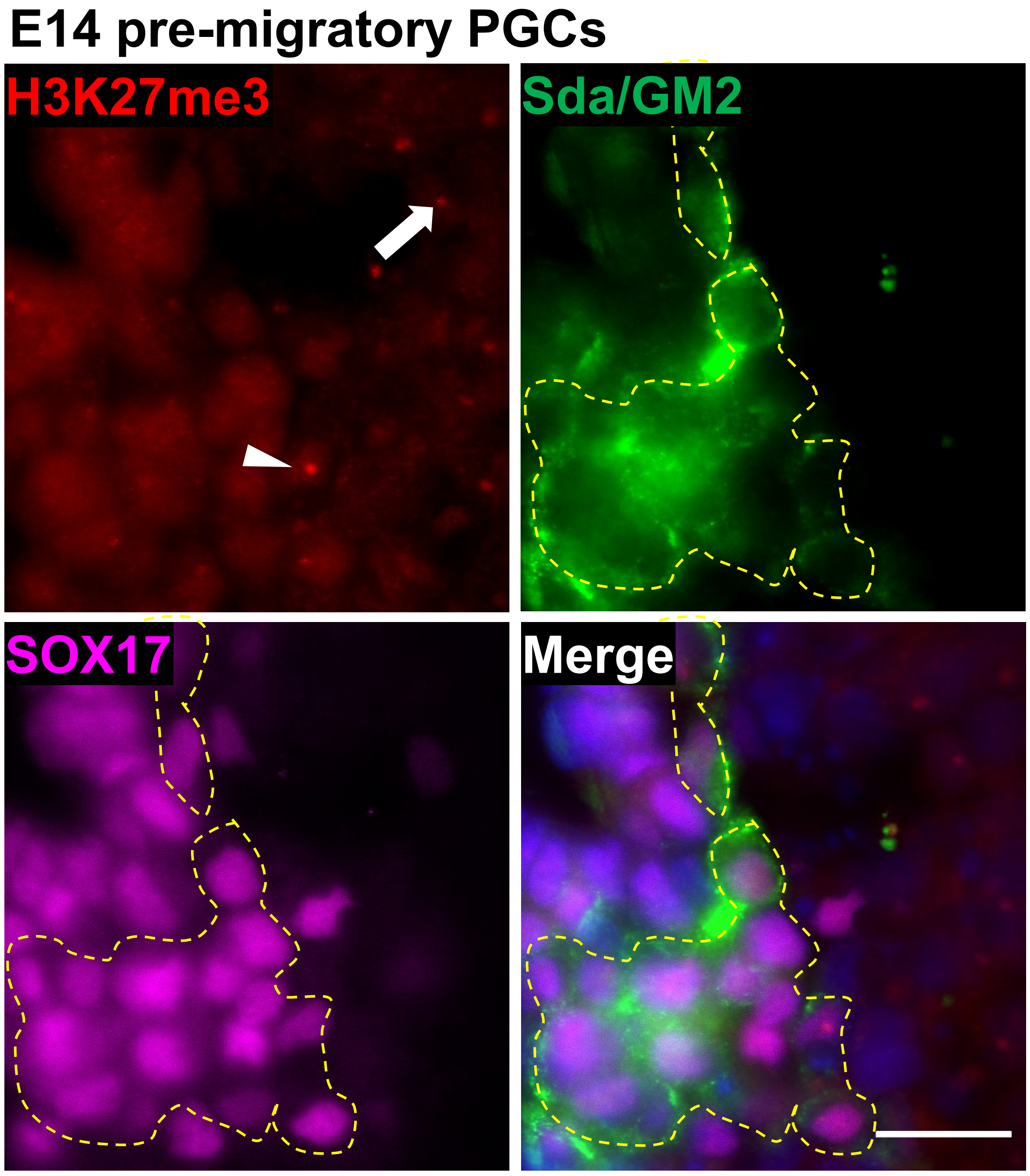

D

	No H3K27me3 spot	Strong H3K27me3 spot	Ambiguous
No. of E17 PGC (%)	96 (72)	19 (14.5)	18 (13.5)
No. of E25 PGC (%)	122 (73)	12 (7.2)	33 (19.8)

Figure S4. Extensive X chromosome Reactivation in Pre-migratory pPGCs. Related to Figure 4.
(A) Sum expression of (top) all Y-chromosome genes, (middle) single-copy Y-chromosome genes and (bottom) all X-chromosome genes.
(B) Bootstrap of X:A ratio of E14 somatic cells, E14 PGCs and E31 PGCs. Each dot represents one cell. P value: pairwise Wilcoxon test.
(C) Immunofluorescence of H3K27me3 in E14 PGC cluster. Yellow circle indicates PGC. Xi-associated H3K27me3 are detected in somatic cells (arrow) and some pPGCs (arrowhead). Scale bar: 20μm.
(D) Quantification of the number of H3K27me3 spots in E17 and E25 pPGC.

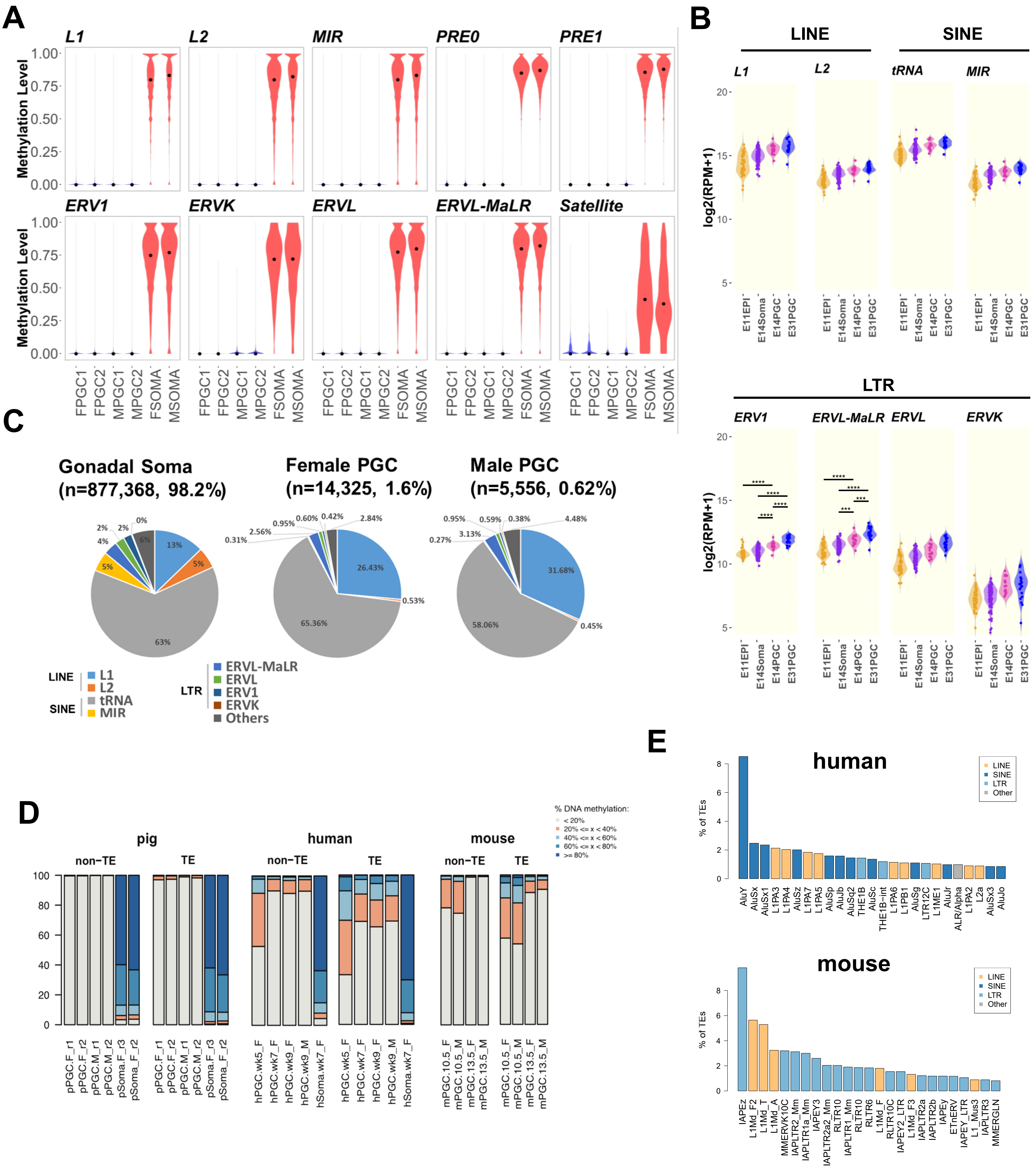

Figure S5. Level of methylation in Wk5 (E35) gonadal pPGCs revealed by BSSeq. Related to Figure 5.
(A) Violin plots showing CpG methylation levels at different repetitive elements.
(B) Expression profiles of major TE families in E11 epiblast, E14 somatic cells, E14 and E31 PGCs. p value. * $p < 0.05$; ** $p < 0.01$; *** $p < 0.001$; **** $p < 0.0001$ by pairwise Wilcoxon test.
(C) Distribution of major TE families that retain partial methylation (≥10%) in pig gonadal soma and PGCs. N: indicates the number of TEs that retain partial methylation, followed by the percentage of those partially methylated TEs among all TEs of the families indicated. L1 is overrepresented in pPGCs samples.
(D) Distribution of CpG methylation in non-TE and TE genomic tiles (800nt, with at least 5 CpG with 1x coverage).
(E) Distribution of TE families that overlap with TE-rich escapees in Wk7-9 hPGCs and E13.5 mPGCs.
See also Table S6**.**

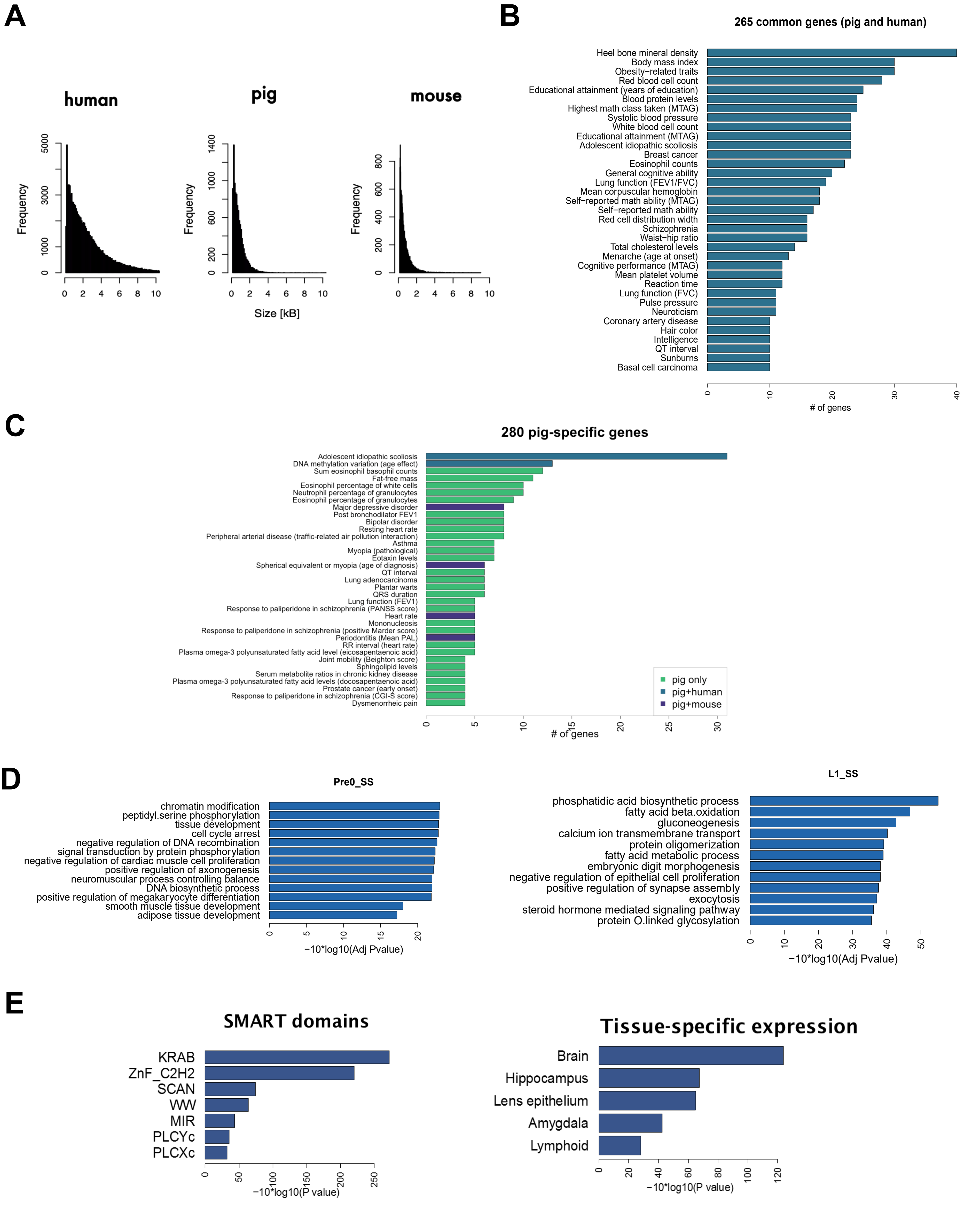

Figure S6. DNA demethylation escapees show common and distinct features between mammals. Related to Figure 6.
(A) Size distribution of demethylation-resistant loci in human, pig, and mouse.
(B) Enrichment analysis for GWAS catalogue terms showing disease and traits associated with conserved TE-poor escapee genes between pig and human.
(C) Enrichment analysis of GWAS catalogue terms for pig-specific TE-poor escapee genes.
(D) GO term enrichment analysis of genes overlapping demethylation-resistant pig-specific TE, Pre_0SS and L1_SS.
(E) Enrichment analysis for SMART domains and tissue-specific expression (UniProt UP_TISSUE) of TE-poor escapee genes shared by at least two species (human, mouse, pig).

Table S8. Primers and Oligonucleotides used. Related to STAR Methods.

Application	Sequence	Reference
scRNASeq.	**TSO** (5′-AAGCAGTGGTATCAACGCAGAGTACATrGrG+G-3′)	Picelli et al., 2014
	**Oligo-dT30VN** (5′–AAGCAGTGGTATCAACGCAGAGTACT30VN-3′)	
	**ISPCR oligo** (5′-AAGCAGTGGTATCAACGCAGAGT-3′)	
PBAT	**BioPEA2N4**: 5'-[btn] CTACACGACGCTCTTCCGATCTNNNNNNN-3'	Clark et al., 2017
	**Rev_N6_PE** (5'-TGCTGAACCGCTCTTCCGATCTNNNNNN-3'	
	**PE 1.0** (5'-AATGATACGGCGACCACCGAGATCTACACTCTTTCCCTACACGACGCTCTTCCGATC*T-3')	
	**iPCR Tag** (5'-CAAGCAGAAGACGGCATACGAGATAACGTGATGAGATCGGTCTCGGCATT CCTGCTGAACCGCTCTTCCGATC*T-3')	
Sexing Pig embryos	**AMEL-F** (5'-CRCMTTCATTGAYAATTCAC-3')	Sembon et al., 2008
	**AMEL-R** 5'-CCAGAGGTTGTAACCTTACAG-3')	
Sexing human embryos	**SRY-F** (5'-TGAACGCATTCATGGTGTGGT-3')	Bryja and Konecny, 2003
	**SRY-R** (5'- AATCTCTGTGCCTCCTGGAA-3')	
